# Navigating outpatient care of patients with type 2 diabetes after hospital discharge - a qualitative longitudinal study

**DOI:** 10.1186/s12913-024-10959-4

**Published:** 2024-04-17

**Authors:** Léa Solh Dost, Giacomo Gastaldi, Marcelo Dos Santos Mamed, Marie P. Schneider

**Affiliations:** 1 School of Pharmaceutical Sciences, University of Geneva, Geneva, Switzerland; 2 Institute of Pharmaceutical Sciences of Western Switzerland, University of Geneva, Geneva, Switzerland; 3 Division of Endocrinology, Diabetes, Hypertension and Nutrition, Department of Medicine, Geneva University Hospitals, Geneva, Switzerland; 4 Institute of Psychology and Education, University of Neuchatel, Neuchâtel, Switzerland; 5 Institute of Psychology, University of Lausanne, Lausanne, Switzerland

**Keywords:** Type 2 diabetes mellitus, Outpatient care, Hospital discharge, Qualitative research

## Abstract

**Background:**

The transition from hospital to outpatient care is a particularly vulnerable period for patients as they move from regular health monitoring to self-management. This study aimed to map and investigate the journey of patients with polymorbidities, including type 2 diabetes (T2D), in the 2 months following hospital discharge and examine patients’ encounters with healthcare professionals (HCPs).

**Methods:**

Patients discharged with T2D and at least two other comorbidities were recruited during hospitalization. This qualitative longitudinal study consisted of four semi-structured interviews per participant conducted from discharge up to 2 months after discharge. The interviews were based on a guide, transcribed verbatim, and thematically analyzed. Patient journeys through the healthcare system were represented using the patient journey mapping methodology.

**Results:**

Seventy-five interviews with 21 participants were conducted from October 2020 to July 2021. The participants had a median of 11 encounters (min–max: 6–28) with HCPs. The patient journey was categorized into six key steps: hospitalization, discharge, dispensing prescribed medications by the community pharmacist, follow-up calls, the first medical appointment, and outpatient care.

**Conclusions:**

The outpatient journey in the 2 months following discharge is a complex and adaptive process. Despite the active role of numerous HCPs, navigation in outpatient care after discharge relies heavily on the involvement and responsibilities of patients. Preparation for discharge, post-hospitalization follow-up, and the first visit to the pharmacy and general practitioner are key moments for carefully considering patient care. Our findings underline the need for clarified roles and a standardized approach to discharge planning and post-discharge care in partnership with patients, family caregivers, and all stakeholders involved.

**Supplementary Information:**

The online version contains supplementary material available at 10.1186/s12913-024-10959-4.

## Background

Care transition is defined as “the movement patients make between healthcare practitioners and settings as their condition and care needs change in the course of a chronic or acute illness” [1]. The transition from hospital to outpatient care is a particularly vulnerable period for patients as they move from a medical environment with regular health monitoring to self-management, where they must implement a large amount of information received during their hospital stay [2–6]. This transition period can be defined as “the post-hospital syndrome,” which corresponds to a transient period of vulnerability (e.g., 30 days) for various health problems, such as stress, immobility, confusion, and even cognitive decline in older adults, leading to complications [7]. Furthermore, discharged patients may experience a lack of care coordination, receive incomplete information, and inadequate follow-ups, leading to potential adverse events and hospital readmissions [8–10].

People with type 2 diabetes mellitus (T2D) represent a high proportion of hospitalized patients, and their condition and medications are associated with a higher rate of hospital readmission [11–13]. Moreover, T2D is generally associated with multiple comorbidities. This complex disease requires time-consuming self-management tasks such as polypharmacy, adaptations of medication dosages, diet, exercise, and medical follow-up, especially during care transition [14–16].

Various interventions and practices, such as enhanced patient education, discharge counseling, and timely follow-up, have been studied to improve care transition for patients with chronic diseases; however, they have shown mixed results in reducing costs and rehospitalization [17–20]. In addition, patient perspectives and patient-reported outcomes are rarely considered; however, their involvement and monitoring are essential for seamless and integrated care [21, 22]. Care integration, an approach to strengthening healthcare systems in partnership with people, focuses on patient health needs, the quality of professional services, and interprofessional collaboration. This approach prevents care fragmentation for patients with complex needs [23, 24]. Therefore, knowledge of healthcare system practices is essential to ensure integrated, coordinated, and high-quality care. Patient perspectives are critical, considering the lack of literature on how patients perceive their transition from hospital to autonomous care management [25, 26].

Patients’ journeys during hospitalization have been described in the literature using various methods such as shadowing, personal diaries, and interviews; however, patients’ experiences after hospital discharge are rarely described [26, 27]. Jackson et al. described the complexity of patient journeys in outpatient care after discharge using a multiple case study method to follow three patients with chronic obstructive pulmonary disease from hospitalization to 3 months post-discharge [26]. The literature does not provide an in-depth understanding of the experiences of patients with comorbidities during care transition upon hospital discharge. The assumption about the patient journey after discharge is that multiple and multi-professional encounters will ensure the transition of care from hospitalization to self-management, but often without care coordination.

This study aimed to investigate the healthcare trajectories of patients with comorbidities, including T2D, during the 2 months following hospital discharge and to examine patients’ encounters with healthcare professionals (HCPs).

## Methods

While this article focuses on patients’ journeys to outpatient care, another article describes and analyzes patients’ medication management, knowledge, and adherence [28]. This study followed the Consolidated Criteria for Reporting Qualitative Research (COREQ).

### Study design and population

A qualitative longitudinal research approach was adopted, with four individual semi-structured interviews over 2 months after discharge (approximately 3, 10, 30, and 60 days after discharge) that took place at home, by telephone, secured video call, or at the university at the participant’s convenience. Participants were recruited during hospitalization. The inclusion criteria were patients with T2D, with at least two other comorbidities, at least one medication change during hospitalization, hospitalization duration of at least 3 days, and those who returned home after discharge and self-managed their medications. A family caregiver could also participate in the interviews alongside to participants.

### Researcher characteristics

All the researchers were trained in qualitative studies. The ward diabetologist and researcher (GG) who enrolled the patients in the study participated in most participants’ care during hospitalization. LS (Ph.D. student and community pharmacist) was unknown to participants and presented herself during hospitalization as a “researcher” rather than a pharmacist to avoid any risk of influencing participants’ answers. MS is a professor in pharmacy, whose research focuses on medication adherence in chronic diseases and aims at better understanding this behavior and its consequences for patients and the healthcare system. MDS is a researcher, linguist, and clinical psychologist, with a particular interest in patients living with chronic conditions such as diabetes and a strong experience in qualitative methodology and verbal data analysis.

### Data collection

The interviews were based on four semi-structured interview guides based on existing frameworks and theories: the World Health Organization’s five dimensions for adherence, the Information-Motivation-Behavioral Skills model, and the Social Cognitive Theory [29–31]. For in-depth documentation of participants’ itinerary in the healthcare system, the interview guides included questions on the type, reason, and moment of the HCP’s encounters and patient relationships with HCPs. Interview guides are available in Supplementary File 1. During the development phase of the study, the interview guides were reviewed for clarity and validity and adapted by two patient partners from the Geneva University Hospitals’ Patient Partner Platform for Research and Patient and Public Involvement. Thematic saturation was considered reached when no new code or theme emerged and new data repeated previously coded information [32]. Sociodemographic and clinical data were collected from hospital databases and patient questionnaires. The interviews were audio-recorded, anonymized, and transcribed verbatim.

### Data analysis

The sociodemographic and clinical characteristics were descriptively analyzed. Transcriptions were double-coded until similar codes were obtained, and thematic analysis, as described by Braun and Clarke [33, 34], was used in a systematic, iterative, and comparative manner. A patient journey mapping methodology was used to illustrate the trajectories of each participant and provide a comprehensive understanding of their experiences. Patient journey mapping is a visual method adapted from the marketing industry that is increasingly used in various health settings and contexts to illustrate and evaluate healthcare services and patient experiences [35]. In this analysis, we used the term “healthcare professionals” when more than one profession could be involved in participants’ healthcare. Otherwise, when a specific HCP was involved, we used the designated profession (e.g. physicians, pharmacists).

## Results

### A. Participants description

Twenty-one participants were interviewed between October 2020 and September 2021, generating 75 interviews. All participants took part in Interview 1, 19 participants in Interview 2, 16 participants in Interview 3 and 19 participants in Interview 4, with a median duration of 41 minutes (IQR: 34-49) per interview. Interviews 1,2,3 and 4 took place respectively 5 days (IQR: 4-7), 14 days (13-20), 35 days (33-38), and 63 days (61-68) after discharge. Nine patients were newly diagnosed with T2D, and 12 had a previous diagnosis of T2D, two of whom were untreated. Further information on participants is described in Table 1. The median number of comorbidities was six (range: 3–11), and participants newly diagnosed with diabetes tended to have fewer comorbidities (median: 4; range: 3–8). More detailed information regarding sociodemographic characteristics and medications has been published previously [28].

**Table 1:** Participants characteristics

**Demographics**	**N (%)**
Participants	21
Age (years), median (IQR)	63 (59–73)
Gender,	
- Men	12 (57%)
- Women	9 (43%)
Reasons for hospitalization	
- Type 2 diabetes	9 (43%)
- Myocardial infarction	4 (19%)
- Other cardiac reasons	5 (24%)
- Other reasons	3 (14%)
Length of hospitalization stay (days), median (IQR)	8 (7–14)
Medications at discharge, median (IQR)	9 (7–12)
Patients treated with insulin	10 (47%)
Comorbidities, median (IQR)	6 (4–7)
Type 2 diabetes diagnosis,	
- Newly diagnosed (< 6 months or during hospitalization)	9 (43%)
- Diagnosed but not treated	2 (10%)
- > 6 months	10 (47%)
Patients hospitalized in the previous 6 months	5 (24%)

### B. Journey mappings

**Fig. 1:**
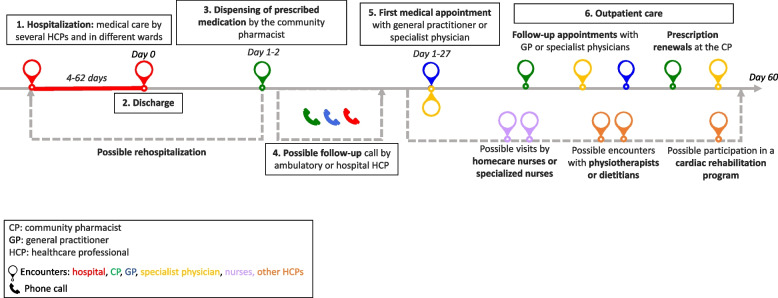
Generic patient journey mapping from hospitalization to two months after discharge

Generic patient journey mapping, presented in Fig. 1, summarizes the main and usual encounters participants had with their HCPs during the study period. Generic mapping results from all individual patient journey mappings from discharge to 2 months after discharge are available in Supplementary File 2.

**Fig. 2:**
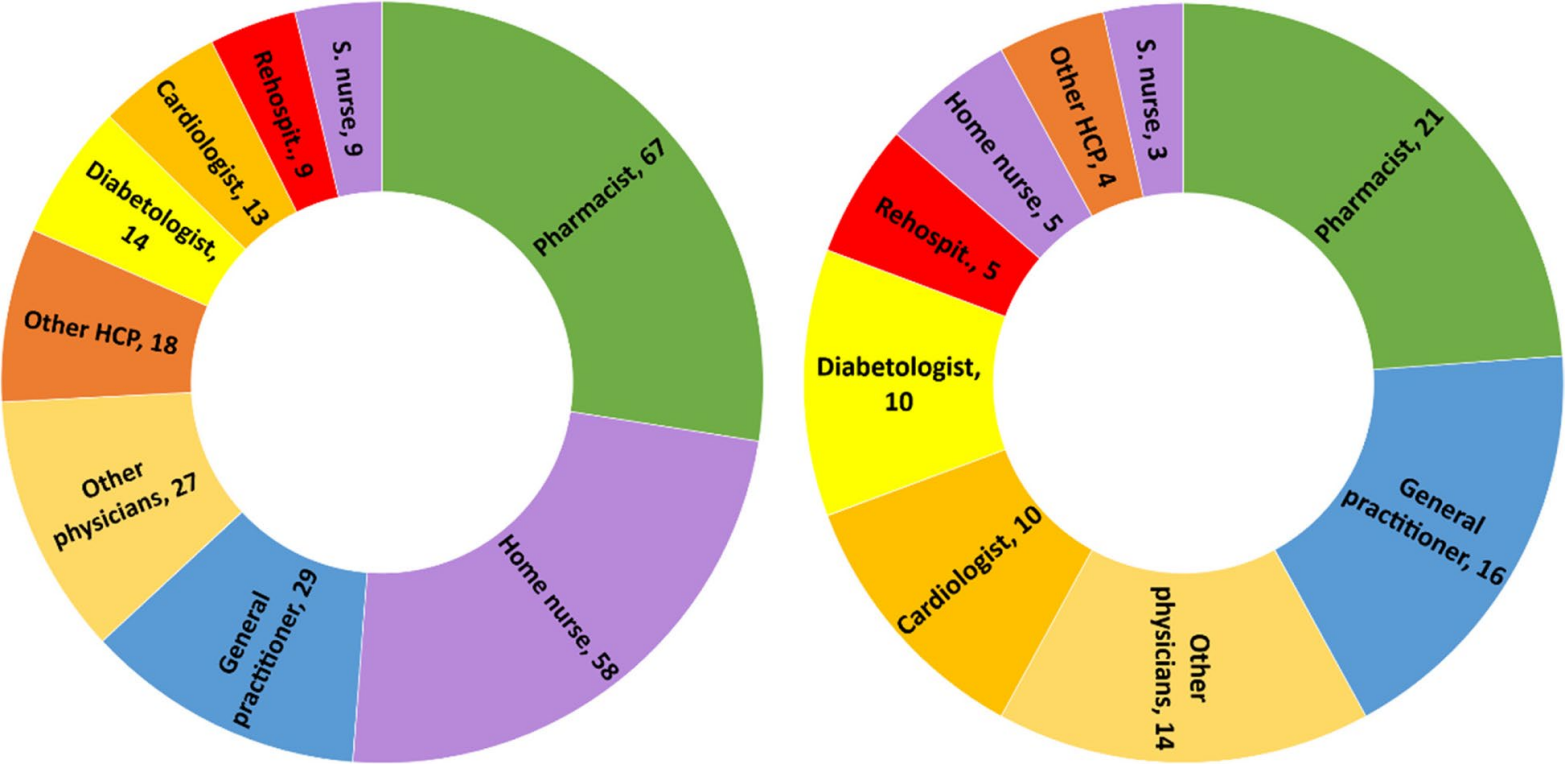
Healthcare professionals seen by participants during the study period. left: n=cumulative encounters; right: n=encountered at least once. Abbreviation: S.nurse: specialized nurse; Other physicians: ophthalmologists, neurologists, hematologists, immunologists, addictologists; other HCP: physiotherapists, dietitians, massage therapist

During the 2 months following discharge, the participants had a median number of 10 (range: 6–28) encounters with HCPs. The HCPs met by participants are represented in Fig. 2. All participants visited their pharmacists at least once, and 16 of the 21 participants met their general practitioners (GPs) at least once. Five participants received home care assistance, four went to an outpatient cardiac rehabilitation program, and five were readmitted during the study period.

The first HCP encountered was at the community pharmacy on the same day or day after discharge, except for one participant who did not pick up her medication. The first medical appointment with a physician occurred between days 1 and 27 after discharge (median: 8; IQR: 6-14).

Participants newly diagnosed with diabetes had a closer follow-up after discharge than participants with a former diagnosis of T2D (median: 7; IQR: 6–10 vs median: 9; IQR: 5–19), fewer encounters with HCPs (median: 8; IQR: 7–10 vs. 11; IQR: 8–17), and fewer comorbidities (median: 4; IQR: 4–7 vs. 7; IQR: 5–9). Most participants newly diagnosed with T2D or receiving insulin treatment benefited from either a follow-up call, home visit by a nurse, or diabetes care appointment.

### C. Qualitative analysis

Transcripts were analyzed longitudinally and categorized into six key steps based on the verbal data. These key steps, shown in Fig. 1, represent the identified thematic categories and refer to the following elements: 1. Hospitalization, 2. Discharge, 3. Dispensing of prescribed medications at the pharmacy, 4. Possible follow-up call, 5. First medical appointment, and 6. Outpatient care.

1. Hospitalization: hospital constraints and care organization

Most participants thought they had benefited from adequate medical care by committed and attentive HCPs but highlighted different constraints and gaps. Some participants noted constraints related to the hospital environment, such as loss of autonomy during their stay, lack of privacy, and the large number of hospital staff encountered. This resulted in participants repeating the same information several times, causing frustration, misunderstanding and a lack of coordination for some participants:

“Twenty or thirty staff members come in during the day! So, it's hard to keep track of [what] is being said or done. The best thing for me [...] would be to have clear information from just one person.” Participant 8; interview 1 (P18.1)

Participants had different opinions on the hospital’s care organization. Some participants found that care coordination between the wards was well-organized. In contrast, others highlighted poor coordination and communication between the hospital wards, resulting in long waiting times, care fragmentation, and contradictory or unclear information. Some participants felt that they did not benefit from comprehensive and integrated care and that the hospital staff focused on the cause of their hospitalization, neglecting other comorbidities:

“They were not interested [in my diabetes and my sight]. I was there for the heart and that was where [my care] stopped.” P17.1

Patients’ involvement in decision-making regarding medical care varied. Some participants were involved in their care and took part in medical decisions. Written information, adequate communication, and health professionals’ interest in patients were highlighted by some participants:

“They took the information sheet and they explained everything to me. They didn't just come once; they came several times to explain everything to me.” P5.1

Other participants found the information difficult to understand, particularly because of their fatigue and because the information was provided orally.

2. Discharge: an unclear process

The discharge process was unclear for patients who could not identify a specific related outpatient medical visit or a key step that summarized their hospital stay and prepared them for discharge:

“Well, there's no real preparation [for discharge]. I was waiting for them to give me the go-ahead so I could go home, that’s all...” P7.4

For some participants, outpatient care follow-up was organized before discharge by the hospital team (generally by making an appointment with the patient’s GP before discharge), whereas others had no post-discharge follow-up scheduled during their hospitalization. Approximately half of the participants refused follow-ups during their hospitalization, such as home care services provided by a nurse, or a rehabilitation hospital stay. The main reason for this refusal was that patients did not perceive the need for follow-up:

“It's true that I was offered a lot of services, which I turned down because I didn't realize how I would manage back at home.” P22.2

3. Dispensing prescribed medications by the community pharmacist: the first HCP seen after discharge

On behalf of half the participants, a family caregiver went to the usual community or hospital outpatient pharmacy to pick up the medications. The main reasons for delegation were tiredness or difficulty moving. In some cases, this missed encounter would have allowed participants to discuss newly prescribed medications with the pharmacist:

“[My husband] went to get the medication. And I thought afterward, […] that I could have asked [the pharmacist]: “But listen, what is this medication for?” I would have asked questions” P2.3

Participants who met their pharmacist after hospital discharge reported a range of pharmaceutical practices, such as checking the prescribed medication against medication history, providing information and explanations, and offering services such as the preparation of pillboxes. For some, the pharmacists’ work at discharge did not differ from regular prescriptions, whereas others found that they received further support and explanations:

“She took the prescription […] checked thoroughly everything and then she wrote how, when, and how much to take on each medication box. She managed it very well and I had good explanations.” P20.3

Some participants experienced problems with generic substitution, the unavailability of medications, or dispensing errors, complicating their journey through the healthcare system.

4. Possible follow-up call by HCP: an unsystematic practice

Some participants received a call from their GP or hospital physician a few days after discharge to check their health or answer questions. These calls reassured participants and their caregivers, who knew they had a point of contact in case of difficulty. Occasionally, participants received calls from their community pharmacists to ensure proper understanding and validate medication changes issued during hospitalization. Some participants did not receive any calls and were disappointed by the lack of follow-up:

“There is no follow-up! Nobody called me from the hospital to see how I was doing […]” P8.2

5. First medical appointment: a key step in the transition of care

The first medical appointment was made in advance by the hospital staff or the patient after discharge. For some participants, this first appointment did not differ from usual care. For most, it was a crucial appointment that allowed them to discuss their hospitalization and new medications and organize their follow-up care. Being cared for by a trusted HCP enabled some patients to feel safe, relieved, and well-cared for, as illustrated by the exchange between a patient and her daughter:

Daughter: When [my mom] came back from the GP, she felt much better [...] It was as if a cork had popped. Was it psychological?

Patient: Maybe… I just felt better.

D: Do you think it was the fact that she paid attention to you as a doctor?

P: She took care of me. She did it in a delicate way. [silence] - P23.2

Some participants complained that their physicians did not receive the hospital discharge letter, making it difficult to discuss hospitalization and sometimes resulting in delayed care.

6. Outpatient care: a multifaceted experience

During the 2 months after hospital discharge, participants visited several physicians (Fig. 2), such as their GP and specialist physicians, for follow-ups, routine check-ups, medical examinations, and new prescriptions. Most participants went to their regular pharmacies to renew their prescriptions, for additional medication information, or for health advice.

Some participants had home care nurses providing various services, such as toileting, care, checks on vital functions, or preparing weekly pill boxes. While some participants were satisfied with this service, others complained that home nurses were unreliable about appointment times or that this service was unnecessary. Some participants were reluctant to use these services:

“The [homecare nurse] makes you feel like you're sick... It's a bit humiliating.” P22.2

Specialized nurses, mostly in diabetology, were appreciated by patients who had dedicated time to talk about different issues concerning diabetes and medication and adapted explanations to the patient’s knowledge. Participants who participated in cardiac rehabilitation said that being in a group and talking to people with the same health problems motivated them to undertake lifestyle and dietary changes:

“In the rehabilitation program, I’m part of a team [of healthcare professionals and patients], I have companions who have gone through the same thing as me, so I’m not by myself. That's better for motivation.” P16.2

6.1  Navigating the outpatient healthcare system: the central role of patients

Managing medical appointments is time-consuming and complex for many participants. Some had difficulty knowing with whom to discuss and monitor their health problems. Others had difficulty scheduling medical appointments, especially with specialist physicians or during holidays. A few participants did not attend some of their appointments because of physical or mental vulnerabilities. Restrictions linked to the type of health insurance coverage made navigating the healthcare system difficult for some participants:

“Some medications weren't prescribed by my GP [...] but by the cardiologist. So, I must ask my GP for a delegation to see the cardiologist. And I have to do this for three or four specialists... Well, it’s a bit of a hassle […] it's not always easy or straightforward”. P11.2

Some participants had financial difficulties or constraints, such as expenses from their hospitalization, ambulance transportation, and medications not covered by their health insurance plans. This led to misunderstandings, stress, and anxiety, especially because some participants could not return to work or, to a lesser extent, because of their medical condition.

To ensure continuity of care, some participants were proactive in their case management, for example, by calling to confirm or obtain further information on an appointment or to ensure information transfer. Written convocations for upcoming medical appointments and tailored explanations helped the participants organize their care. Family caregivers were also key in taking participants to various consultations, reminding them, and managing their medical appointments.

6.2  Information transfer: incomplete and missing information

Information transfer between and within settings was occasionally lacking. Even weeks after hospitalization, some documents were not transmitted to outpatient physicians, sometimes delaying medical care. Some participants reported receiving incomplete, unclear, or contradictory information from different HCPs, sometimes leading to doubts, seeking a second medical opinion, or personal searches for information. A few proactive participants ensured good information transmission by making a copy of the prescription or sending copies of their documents to physicians:

“My GP hasn't received anything from the hospital yet. I’ve sent him the PDF with the medication I take before our appointment […] Yes, It’s the patient that does all the job.” P10.3

6.3  Interprofessional work: a practice highlighted by some participants

Several participants highlighted the interprofessional work they observed in the outpatient setting, especially because they had several comorbidities; therefore, several physicians followed their care:

“My case is very complex! For example, between the cardiologist and the diabetologist, they need to communicate closely because there could be consequences or interactions with the medications I take [for my heart and my diabetes].” P4.2

Health professionals referred their patients to the most appropriate provider for better follow-up (e.g., a nurse specializing in addictology referred a patient to a nurse specializing in diabetology for questions and follow-up on blood sugar levels). Interprofessional collaboration between physicians and pharmacists was noted by some participants, especially for prescription refills or ordering medications.

6.4  Patient-HCPs relationships: the importance of trust

Trust in the care relationship was discussed by the participants regarding different HCPs, especially GPs and community pharmacists. Most participants highlighted the communication skills and active listening of healthcare providers. Knowing an HCP for several years helped build trust and ensure an updated medical history:

“I've trusted this pharmacist for 20 years. I can phone her or go to the pharmacy to ask any question[...] I feel supported.” P3.2

Some participants experienced poor encounters owing to a lack of attentive listening or adapted communication, especially when delivering bad news (new diagnoses or deterioration of health status). Professional competencies were an important aspect of the patient-HCP relationship, and some participants lost confidence in their physician or pharmacist because of inadequate medical or pharmaceutical care management or errors, such as the physician prescribing the wrong medication dosage, the pharmacist delivering the wrong pillbox or the general practitioner refusing to see a patient:

“I think I'll find another doctor… In fact, the day I was hospitalized, I called before to make an appointment with her and she refused to see me […] because I had a fever, and I hadn’t done a [COVID] test.” P6.2

Most participants underlined the importance of their GP because they were available, attentive to their health issues, and had a comprehensive view of their medications and health, especially after hospitalization:

“Fortunately, there are general practitioners, who know everything. With some specialists, the body is fragmented, but my GP knows the whole body.” P14.1

After hospitalization, the GP’s role changed for some participants who saw their GP infrequently but now played a central role.

6.5  Community pharmacist: an indistinct role

Pharmacists and their teams were appreciated by most participants for their interpersonal competencies, such as kindness, availability, professional flexibility, and adaptability to patients’ needs to ensure medication continuity (e.g., extension of the prescription, home delivery, or extending time to pay for medications). The role of community pharmacists varied according to the participants. Some viewed pharmacists as simple salespeople:

“It's like a grocery store. [...] I go there, it's ordered, I take my medication, I pay and I leave.” P23.3

For others, the pharmacist provided medication and advice and was a timely source of information but did not play a central role in their care. For others, the pharmacist’s role is essential for medication monitoring and safety:

“I always go to the same pharmacy […] because I know I have protection: when [the pharmacist] enters the medications in his computer, if two medications are incompatible, he can verify. [...] There is this follow-up that I will not have if I go each time somewhere else.” P10.4

## Discussion

The patient journey mapping methodology, coupled with qualitative thematic analysis, enabled us to understand and shed light on the intricacies of the journey of polypharmacy patients with T2Din the healthcare system after discharge. This provided valuable insights into their experiences, challenges, and opportunities for improvement.

This study highlights the complex pathways of patients with comorbidities by considering the population of patients with T2D as an example. Our population included a wide variety of patients, both newly diagnosed and with known diabetes, hospitalized for T2D or other reasons. Navigating the healthcare system was influenced by the reason for hospitalization and diagnosis. For example, newly diagnosed participants with T2D had a closer follow-up after discharge, participants were more likely to undergo cardiac rehabilitation after infarction, and participants with a former T2D diagnosis were more complex, with more comorbidities and more HCP encounters. Our aim was not to compare these populations but to highlight particularities and differences in their health care and these qualitative data reveal the need for further studies to improve diabetes management during inpatient to outpatient care transition.

The variability in discharge practices and coordination with outpatient care highlights the lack of standardization during and after hospital discharge. Some participants had a planned appointment with their GP before discharge, others had a telephone call with a hospital or ambulatory physician, and some had no planned follow-up, causing confusion and stress. Although various local or national guidelines exist for managing patients discharged from the hospital [36–39], there are no standard practices regarding care coordination implemented in the setting of this study. The lack of local coordination has also been mentioned in other studies [5, 40, 41].

Our results also raise questions about the responsibility gap in the transition of care. Once discharged from the hospital, who is responsible for the patient until their first medical appointment? This responsibility is not clearly defined among hospital and outpatient care providers, with more than 25% of internal medicine residents indicating their responsibility for patients ending at discharge [42, 43]. Importance should be given to clarifying when and who will take over the responsibility of guaranteeing patient safety and continuity of care and avoiding rehospitalization [44].

The first visit with the community pharmacist after discharge and the referring physician were the key encounters. While the role of the GP at hospital discharge is well-defined, the community pharmacist’s role lacks clarity, even though they are the first HCP encountered upon hospital discharge. A meta-analysis showed the added value of community pharmacists and how their active participation during care transition can reduce readmission [18]. A better definition of the pharmacist’s role and integration into care coordination could benefit patient safety during the transition and should be assessed in future studies.

Our findings showed that the time elapsed between discharge and the first medical appointment varied widely (from 1 to 27 days), correlating with findings in the literature showing that more than 80% of patients see their GP within 30 days [45]. Despite the first medical appointment being within the first month after discharge, some patients in our study reported a lack of support and follow-up during the first few days after discharge. Care coordination at discharge is critical, as close outpatient follow-up within the first 7–10 days can reduce hospital readmission rates [46, 47]. Furthermore, trust and communication skills are fundamental components of the patient-HCP relationship, underlined in our results, particularly during the first medical appointment. Relational continuity, especially with a particular HCP who has comprehensive patient knowledge, is crucial when patients interact with multiple clinicians and navigate various settings [48, 49].

Navigating the outpatient healthcare system after discharge was complex for most participants and relied heavily on patient involvement and responsibility. While some participants who received clear information felt more empowered and engaged in their care, others highlighted the difficulty in organizing their care during this vulnerable period. Such difficulties in case management have been described previously [50, 51]. Moreover, services proposed by HCPs (e.g., home assistance) do not always correspond to patient needs and are sometimes refused. This highlights the tension between HCPs’ medical recommendations, priorities, and patient expectations. This tension between medical priorities and patient needs was felt during hospitalization and shaped the 2 months following discharge. HCPs need to assess patient needs and preferences during hospitalization and transition for follow-up services. They must also ensure that the offered services meet at least the most relevant of patients’ perceived needs to improve seamless care and patient safety [52, 53].

Examples of a lack of communication and information transfer were described in our results at different levels among HCPs, between participants or family caregivers, and HCPs, and these findings correlate with the literature [3, 54–56]. Although family caregivers play an important role in supporting patients in the healthcare system, they are also additional interlocutors, leading to missed opportunities for patient-pharmacist interactions when dispensing discharged medication. Therefore, it is paramount to integrate and involve family caregivers in shared decision-making and communicate with patients remotely when they are not present [57].

Opportunities to improve the discharge of patients returning home after discharge without home care are highlighted in this article. Our insights can serve as a valuable foundation for healthcare providers and policymakers seeking to optimize patient experience and quality of care in the post-discharge phase. Different professionals should be integrated into standard practice through guidelines to ensure improved collaboration from hospital discharge to outpatient care. During hospitalization:

1. an appointment should be scheduled with the referring physician shortly after discharge to guarantee continuity of care
2. a hospital discharge interview should be conducted in a systematic way to summarize and securely close the hospitalization
3. the community pharmacist should be informed before the patient’s discharge to prepare and reconcile medications before and after hospitalization

In outpatient care:

4. an in-person or phone encounter with the pharmacy team should be scheduled for the patient and/or caregivers at discharge
5. a contact point (phone number, email, or virtual chat assistant) or scheduled follow-up should be implemented to answer questions and redirect patients before they can meet with the referring physician
6. a long-term and active communication channel between HCPs should be established.

In other countries, several outpatient services are already available for patients discharged home to enhance continuity of care and patient safety after discharge. The telehealth-based Transitional Care Management Programme, a local initiative in a New York hospital, involves contacting discharged patients 24 to 48 hours after discharge to support understanding of discharge instructions, medication access, follow-up appointments, and social needs [58]. The Australian Government has introduced the Transition Care Program that provides short-term care for older people, including social work, nursing support, personal care, and allied health care [59]. In England, the NHS has introduced the Discharge Medicines Service (DMS) in community pharmacies, which aims to improve communication between hospitals and community pharmacies and to ensure that patients understand changes to their medications [60].

### Limitations

This study has several limitations. First, the accuracy of the encounter dates with HCPs, as described by the participants, could not be verified using a second data source (e.g., medical or pharmacy records). Additionally, recall biases cannot be excluded, especially during interviews 3 and 4, which took place at longer intervals (20 days between interviews 2 and 3 and 30 days between interviews 3 and 4). Nevertheless, our findings express a patient's representation of their healthcare system navigation experience. Secondly, these results may not be generalizable to populations with other long-term diseases, even though we recruited patients with different reasons for hospitalization, including age, sex, and comorbidities. In addition, the study region is predominantly an urban area with a high density of HCPs, which may influence patient journeys in the healthcare system. Finally, we excluded patients whose medications were managed by HCPs because these patients might have had different experiences, difficulties, and needs. This exclusion criterion was chosen because our objective was to investigate patients’ medication self-management, as described in another article [28].

## Conclusion

A patient’s journey in the 2 months following discharge is unique for each individual and constitutes a complex and adaptive process. Despite the active role of numerous HCPs, navigation in outpatient care after discharge relies heavily on the involvement and responsibilities of polypharmacy. The findings of this study highlight the need to standardize the approach for discharge planning and post-discharge care in partnership with patients and caregivers. Preparation for discharge, the first visit to the pharmacy, and the first appointment with the GP are key moments for all patients, along with the involvement of other medical and nurse specialists, as needed. Standardizing practices, clarifying responsibilities, integrating community pharmacists during the transition, empowering patients, and enhancing interprofessional communication and collaboration should be explored and implemented to achieve better patient outcomes and a more seamless healthcare journey for individuals transitioning from the hospital to the community.

### Supplementary Information

**Additional file 1.** Interview guides.

**Additional file 2.** Individual patient journey mappings from discharge to 2 months after discharge.

## Data Availability

The qualitative codes in French and anonymized patient datasets are available from the corresponding author on reasonable request. Individual patient journeys are provided in the Supplementary Files.

## Abbreviations

GP: General practitioner
HCP: Healthcare professional
T2D: type 2 diabetes mellitus
