# Supplementary material for: Navigating outpatient care of patients with type 2 diabetes after hospital discharge - a qualitative longitudinal study
Source: BMC Health Serv Res. 2024 Apr 17;24:476. doi: 10.1186/s12913-024-10959-4 (PMC11022398; doi:10.1186/s12913-024-10959-4)
Supplement: Supplementary file 2 — Additional file 2. Individual patient journey mappings from discharge to 2 months after discharge. [file 12913_2024_10959_MOESM2_ESM.pdf]

Additional File 2: individual patient journey mappings from discharge to 2 months post-discharge

insulin treatment on discharge from hospital

Type 2 diabetes diagnosed during hospitalization or within the last 6 months

Encounters: hospital, OCP, CP, GP, specialist physician, other HCPs

Phone call

Important/Main medication changes

Vaccine

AN: addictology nurse

CP: community pharmacist

DN: diabetes nurse

GP: general practitioner

HM: home nurse

OCP: Outpatient community pharmacist

P1 NEW

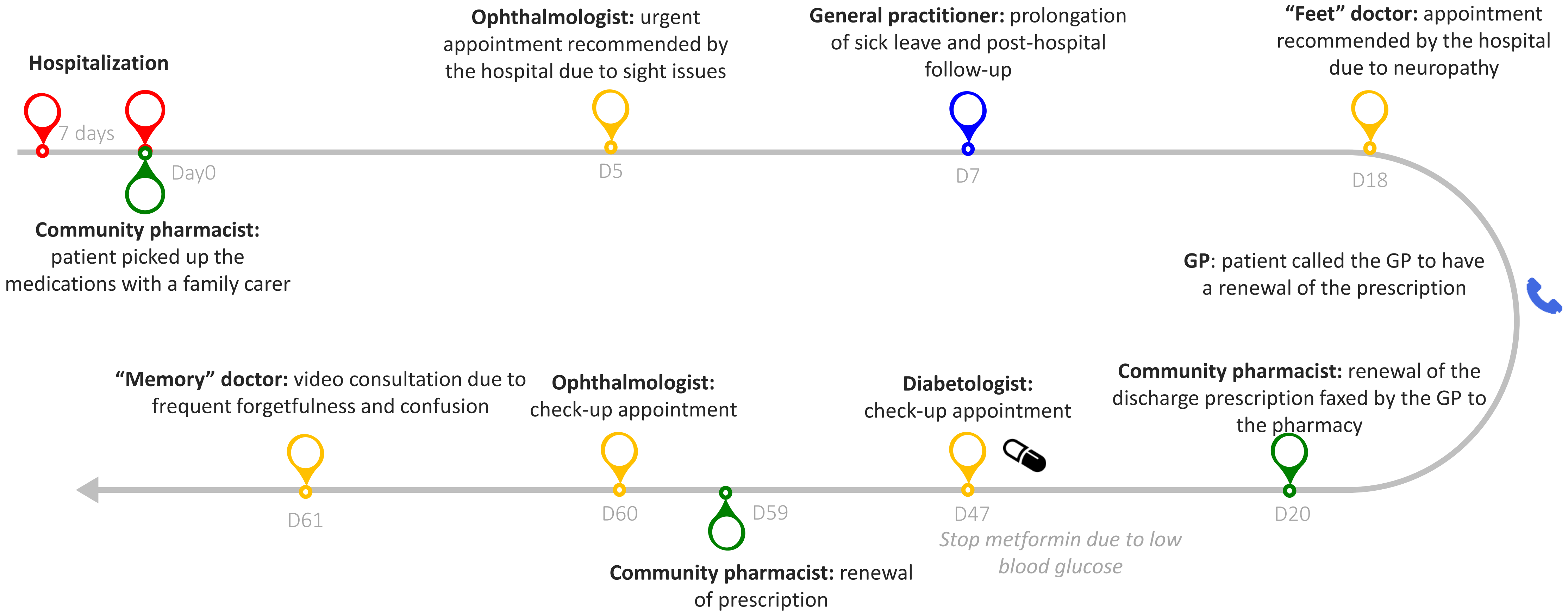
Hospitalization
7 days
Day0
Community pharmacist: patient picked up the medications with a family carer
Ophthalmologist: urgent appointment recommended by the hospital due to sight issues
D5
General practitioner: prolongation of sick leave and post-hospital follow-up
D7
"Feet" doctor: appointment recommended by the hospital due to neuropathy
D18
GP: patient called the GP to have a renewal of the prescription
Community pharmacist: renewal of the discharge prescription faxed by the GP to the pharmacy
D20
Diabetologist: check-up appointment
D47
Stop metformin due to low blood glucose
Community pharmacist: renewal of prescription
D59
Ophthalmologist: check-up appointment
D60
"Memory" doctor: video consultation due to frequent forgetfulness and confusion
D61

P2

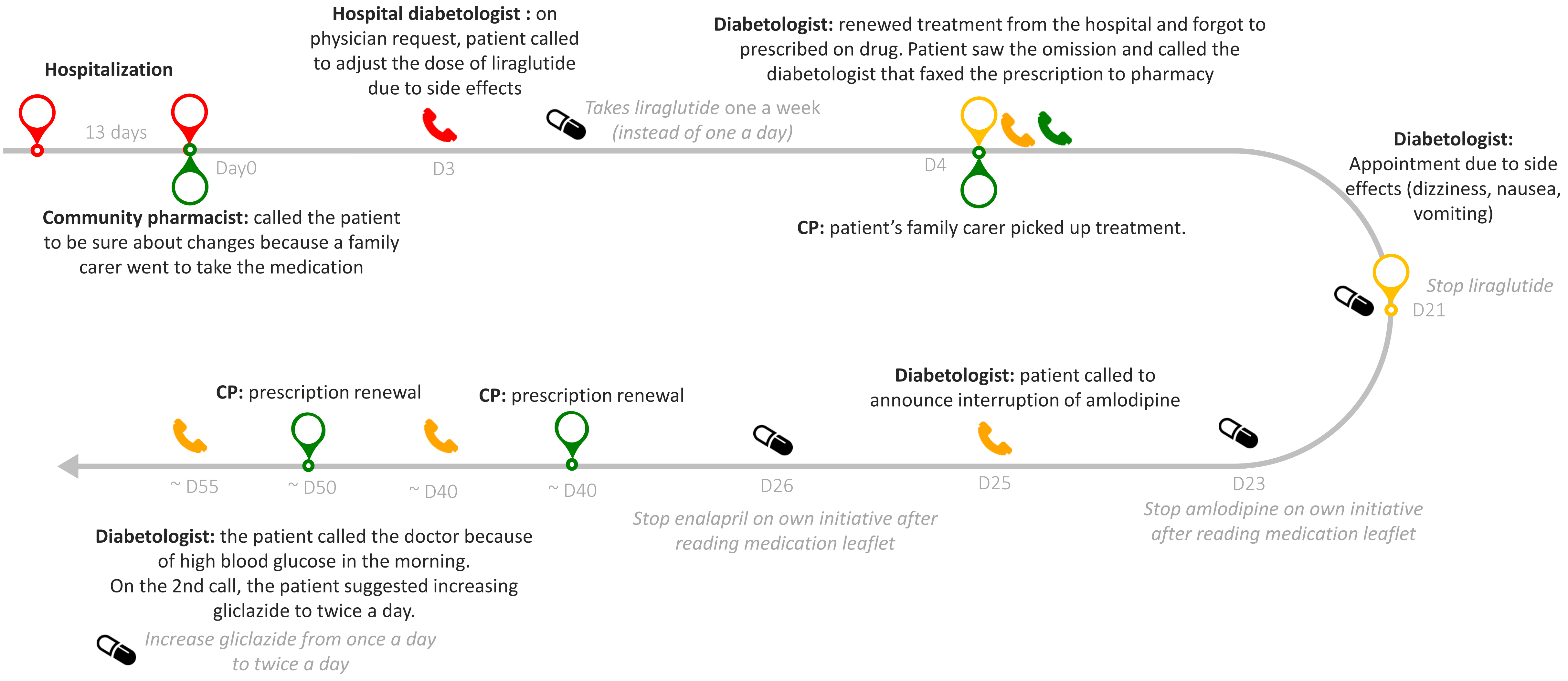

Hospitalization
13 days
Day0
Community pharmacist: called the patient to be sure about changes because a family carer went to take the medication
Hospital diabetologist : on physician request, patient called to adjust the dose of liraglutide due to side effects
D3
Takes liraglutide one a week (instead of one a day)
Diabetologist: renewed treatment from the hospital and forgot to prescribed on drug. Patient saw the omission and called the diabetologist that faxed the prescription to pharmacy
D4
CP: patient's family carer picked up treatment.
Diabetologist: Appointment due to side effects (dizziness, nausea, vomiting)
Stop liraglutide
D21
Stop amlodipine on own initiative after reading medication leaflet
D23
Diabetologist: patient called to announce interruption of amlodipine
D25
Stop enalapril on own initiative after reading medication leaflet
D26
CP: prescription renewal
~ D40
~ D40
CP: prescription renewal
~ D50
~ D55
Diabetologist: the patient called the doctor because of high blood glucose in the morning.
On the 2nd call, the patient suggested increasing gliclazide to twice a day.
Increase gliclazide from once a day to twice a day

P3

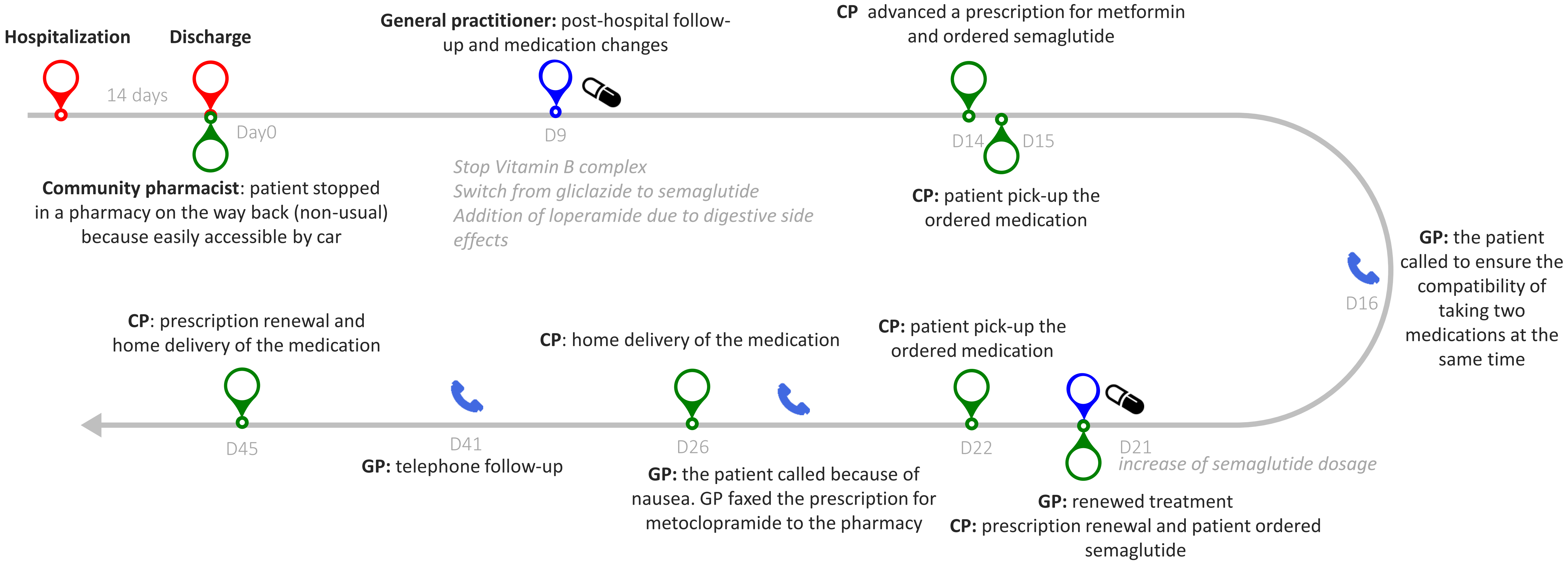
Hospitalization
Discharge
14 days
Day0
Community pharmacist: patient stopped in a pharmacy on the way back (non-usual) because easily accessible by car
General practitioner: post-hospital follow-up and medication changes
D9
Stop Vitamin B complex
Switch from gliclazide to semaglutide
Addition of loperamide due to digestive side effects
CP advanced a prescription for metformin and ordered semaglutide
D14
D15
CP: patient pick-up the ordered medication
D16
GP: the patient called to ensure the compatibility of taking two medications at the same time
CP: patient pick-up the ordered medication
D22
D21
increase of semaglutide dosage
GP: renewed treatment
CP: prescription renewal and patient ordered semaglutide
CP: home delivery of the medication
D26
GP: the patient called because of nausea. GP faxed the prescription for metoclopramide to the pharmacy
D41
GP: telephone follow-up
CP: prescription renewal and home delivery of the medication
D45

P4

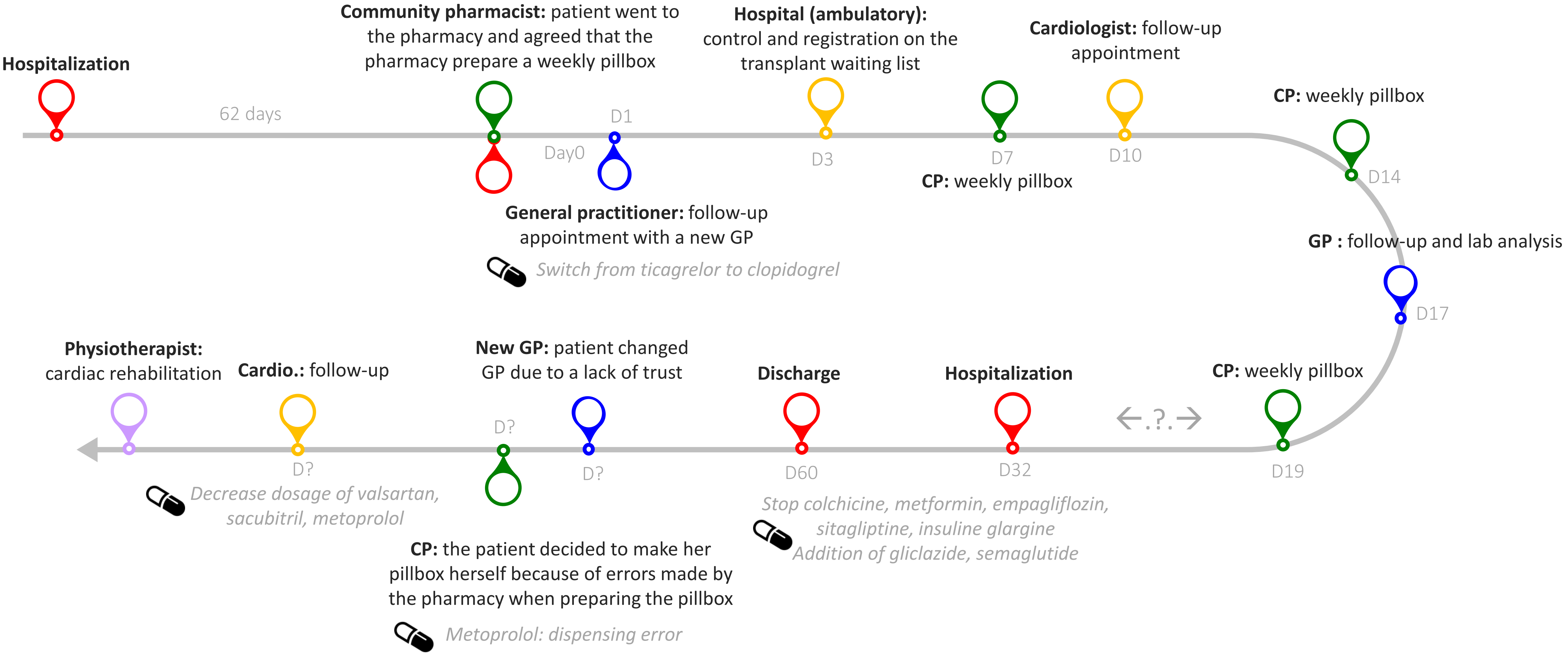

Hospitalization
62 days
Community pharmacist: patient went to the pharmacy and agreed that the pharmacy prepare a weekly pillbox
Day0
D1
General practitioner: follow-up appointment with a new GP
Switch from ticagrelor to clopidogrel
Hospital (ambulatory): control and registration on the transplant waiting list
D3
D7
CP: weekly pillbox
Cardiologist: follow-up appointment
D10
CP: weekly pillbox
D14
GP : follow-up and lab analysis
D17
CP: weekly pillbox
D19
←.?.→
Hospitalization
D32
Discharge
D60
Stop colchicine, metformin, empagliflozin, sitagliptine, insuline glargine
Addition of gliclazide, semaglutide
New GP: patient changed GP due to a lack of trust
D?
D?
CP: the patient decided to make her pillbox herself because of errors made by the pharmacy when preparing the pillbox
Metoprolol: dispensing error
Cardio.: follow-up
D?
Decrease dosage of valsartan, sacubitril, metoprolol
Physiotherapist: cardiac rehabilitation

P5

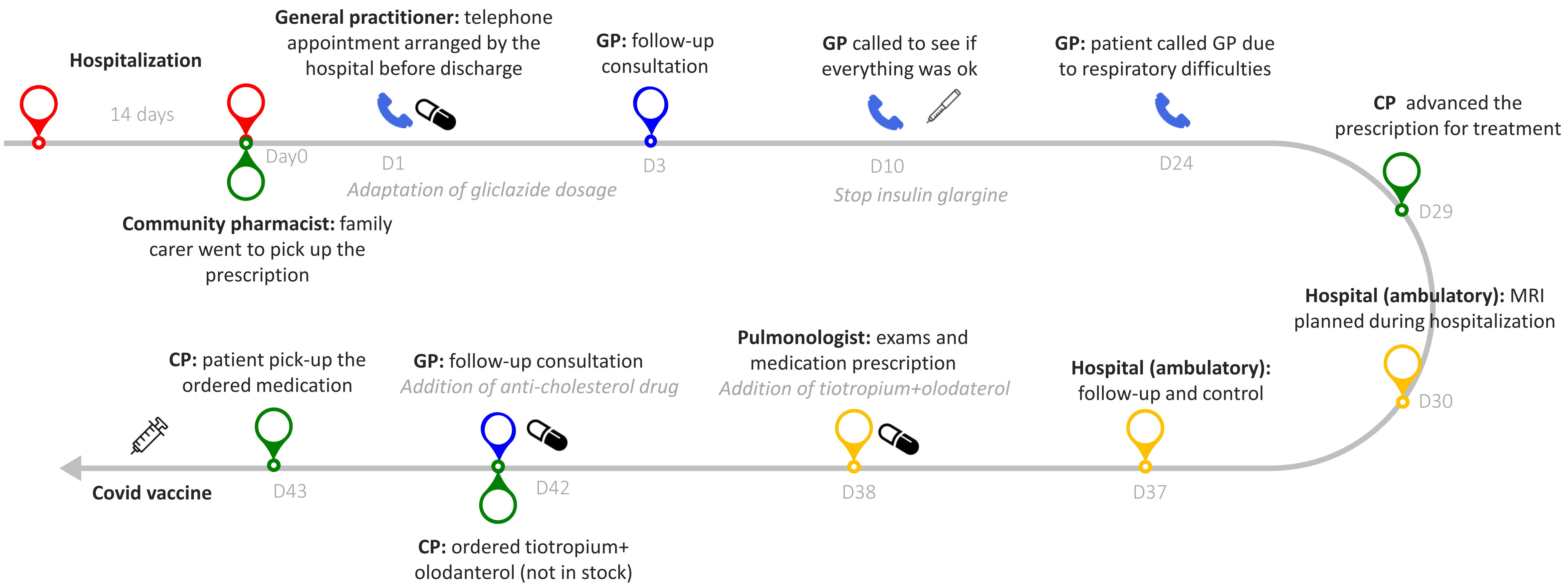

Hospitalization
14 days
Day0
General practitioner: telephone appointment arranged by the hospital before discharge
D1
Adaptation of gliclazide dosage
GP: follow-up consultation
D3
GP called to see if everything was ok
D10
Stop insulin glargine
GP: patient called GP due to respiratory difficulties
D24
CP advanced the prescription for treatment
D29
Community pharmacist: family carer went to pick up the prescription
Hospital (ambulatory): MRI planned during hospitalization
D30
Hospital (ambulatory): follow-up and control
D37
Pulmonologist: exams and medication prescription
Addition of tiotropium+olodaterol
D38
GP: follow-up consultation
Addition of anti-cholesterol drug
D42
CP: ordered tiotropium+ olodanterol (not in stock)
CP: patient pick-up the ordered medication
D43
Covid vaccine

P6

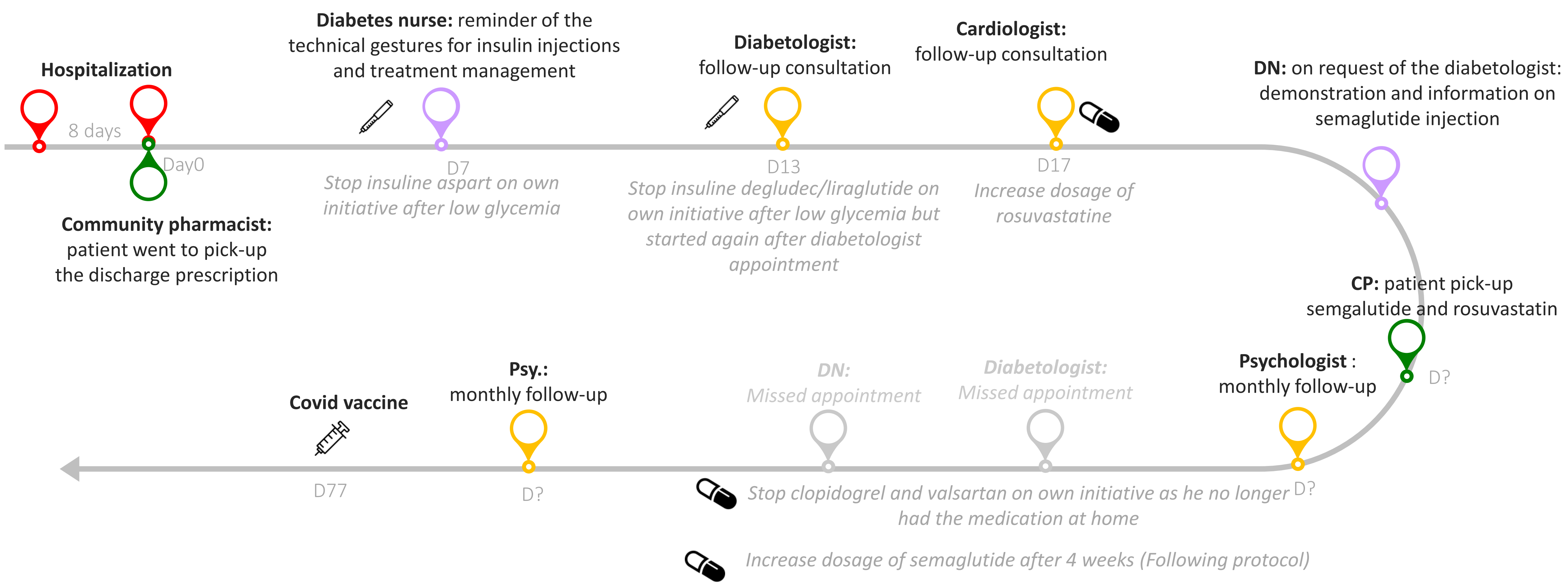
Hospitalization
8 days
Day0
Community pharmacist: patient went to pick-up the discharge prescription
Diabetes nurse: reminder of the technical gestures for insulin injections and treatment management
D7
Stop insuline aspart on own initiative after low glycemia
Diabetologist: follow-up consultation
D13
Stop insuline degludec/liraglutide on own initiative after low glycemia but started again after diabetologist appointment
Cardiologist: follow-up consultation
D17
Increase dosage of rosuvastatine
DN: on request of the diabetologist: demonstration and information on semaglutide injection
CP: patient pick-up semgalutide and rosuvastatin
D?
Psychologist : monthly follow-up
D?
Diabetologist: Missed appointment
DN: Missed appointment
Stop clopidogrel and valsartan on own initiative as he no longer had the medication at home
Increase dosage of semaglutide after 4 weeks (Following protocol)
Psy.: monthly follow-up
D?
Covid vaccine
D77

P7

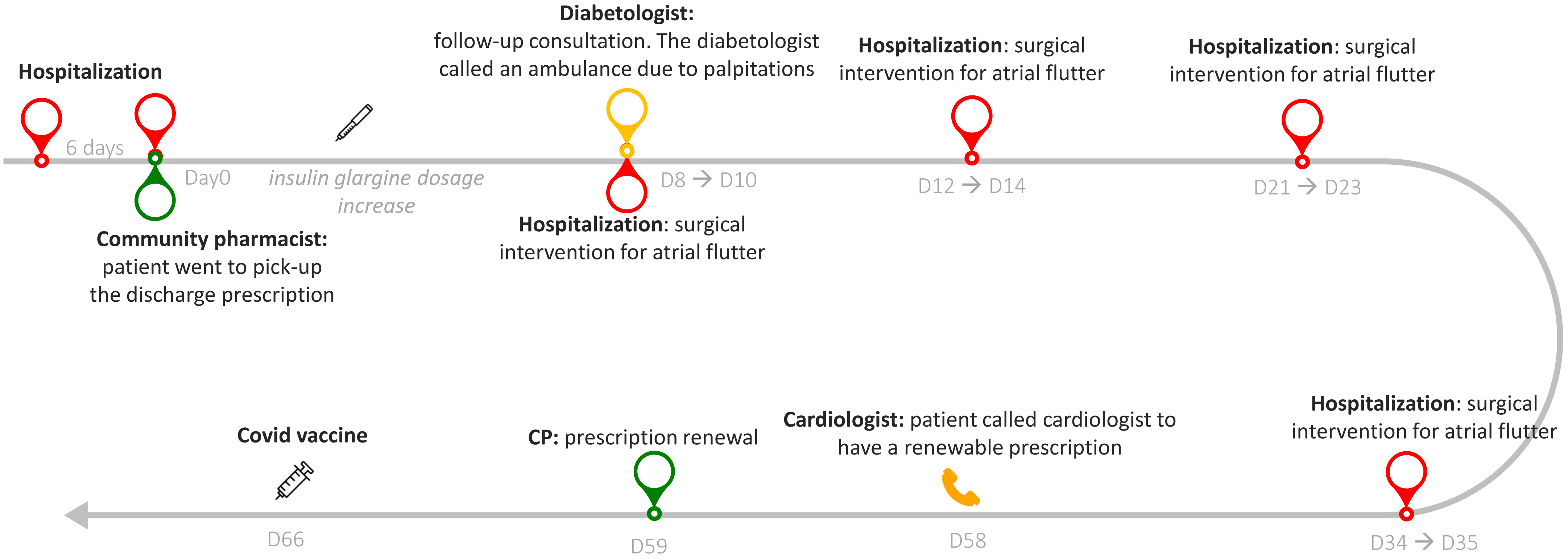

Hospitalization
6 days
Day0
Community pharmacist: patient went to pick-up the discharge prescription
insulin glargine dosage increase
Diabetologist: follow-up consultation. The diabetologist called an ambulance due to palpitations
D8 → D10
Hospitalization: surgical intervention for atrial flutter
Hospitalization: surgical intervention for atrial flutter
D12 → D14
Hospitalization: surgical intervention for atrial flutter
D21 → D23
Hospitalization: surgical intervention for atrial flutter
D34 → D35
Cardiologist: patient called cardiologist to have a renewable prescription
D58
CP: prescription renewal
D59
Covid vaccine
D66

P8

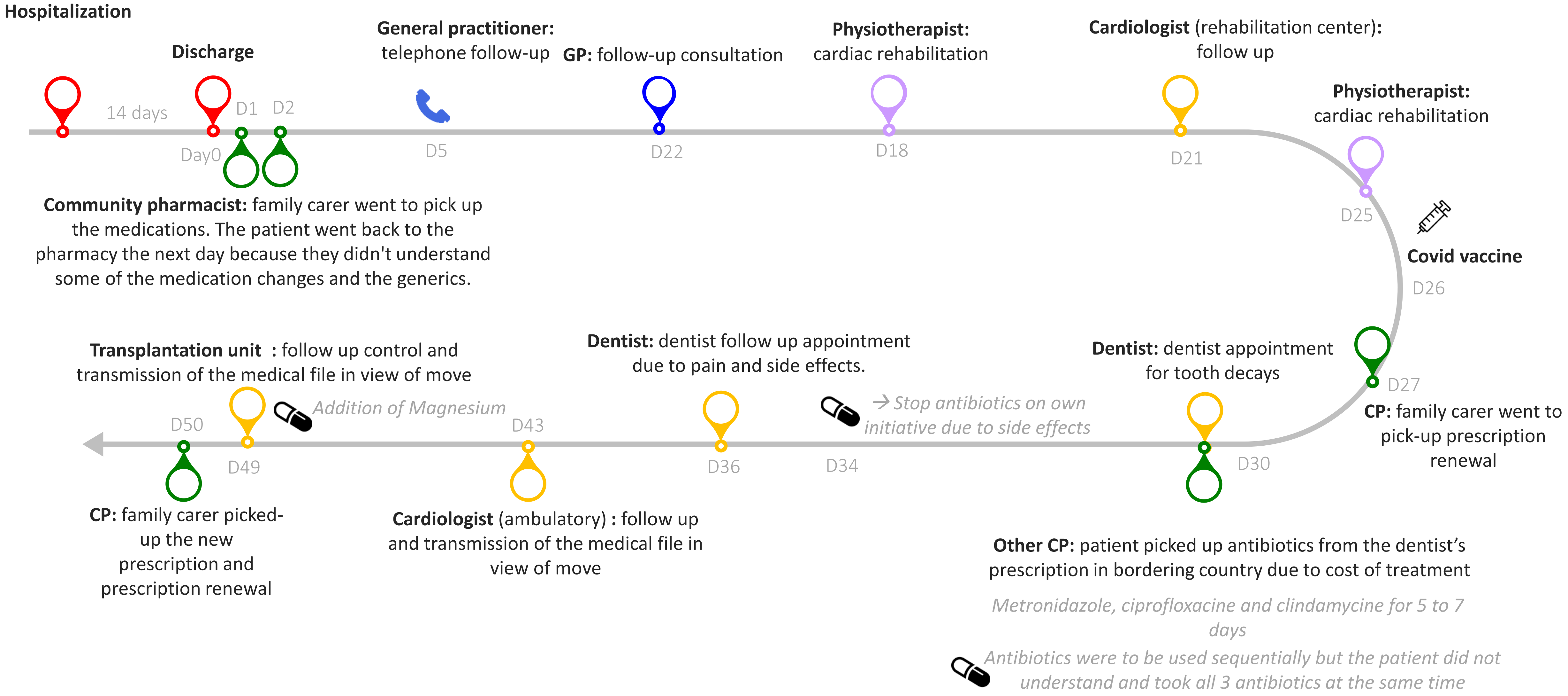

Hospitalization
14 days
Discharge
Day0
D1
D2
General practitioner: telephone follow-up
D5
GP: follow-up consultation
D22
Physiotherapist: cardiac rehabilitation
D18
Cardiologist (rehabilitation center): follow up
D21
Physiotherapist: cardiac rehabilitation
D25
Covid vaccine
D26
Community pharmacist: family carer went to pick up the medications. The patient went back to the pharmacy the next day because they didn't understand some of the medication changes and the generics.
D27
CP: family carer went to pick-up prescription renewal
Dentist: dentist appointment for tooth decays
D30
Other CP: patient picked up antibiotics from the dentist's prescription in bordering country due to cost of treatment
Metronidazole, ciprofloxacine and clindamycine for 5 to 7 days
Antibiotics were to be used sequentially but the patient did not understand and took all 3 antibiotics at the same time
→ Stop antibiotics on own initiative due to side effects
D34
Dentist: dentist follow up appointment due to pain and side effects.
D36
D43
Cardiologist (ambulatory) : follow up and transmission of the medical file in view of move
Addition of Magnesium
D49
Transplantation unit : follow up control and transmission of the medical file in view of move
D50
CP: family carer picked-up the new prescription and prescription renewal

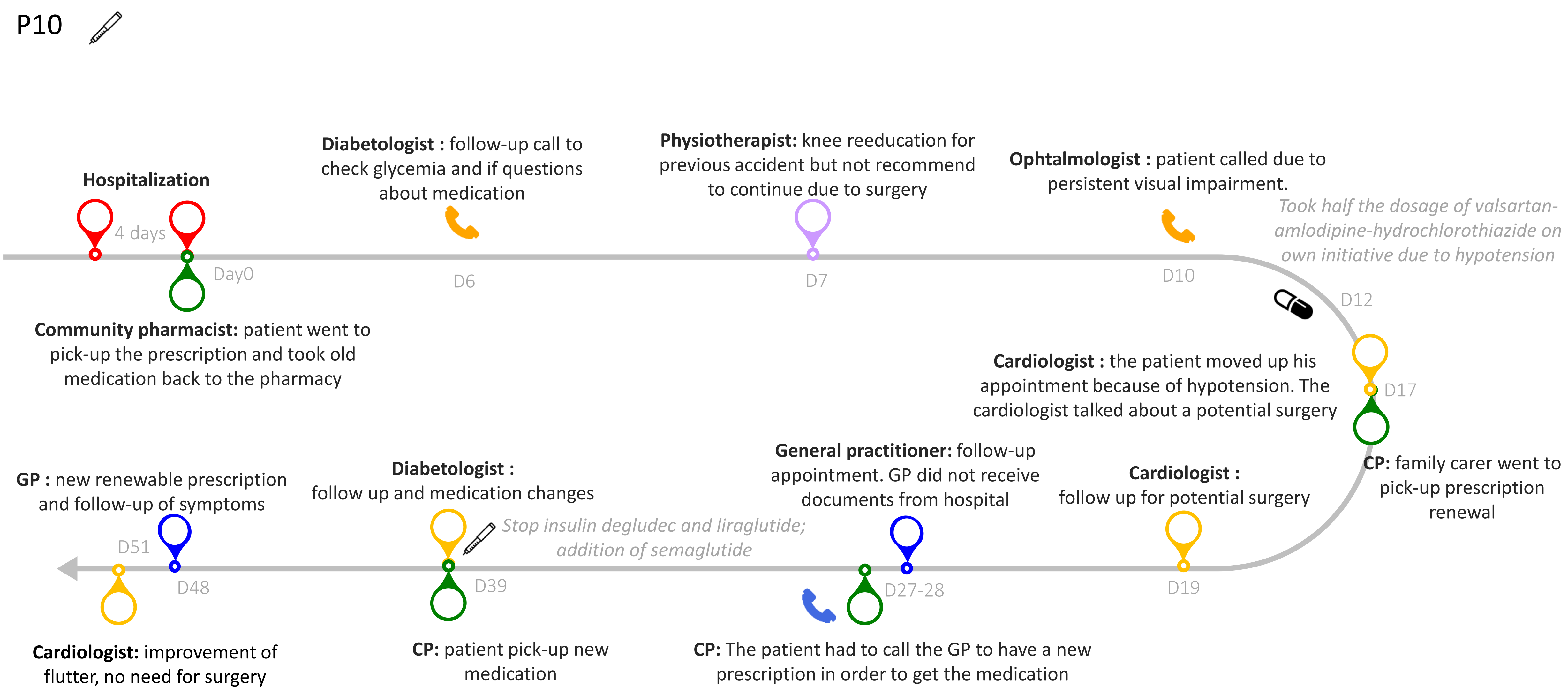

P10
Hospitalization
4 days
Day0
Community pharmacist: patient went to pick-up the prescription and took old medication back to the pharmacy
Diabetologist : follow-up call to check glycemia and if questions about medication
D6
Physiotherapist: knee reeducation for previous accident but not recommend to continue due to surgery
D7
Ophtalmologist : patient called due to persistent visual impairment.
D10
Took half the dosage of valsartan-amlodipine-hydrochlorothiazide on own initiative due to hypotension
D12
Cardiologist : the patient moved up his appointment because of hypotension. The cardiologist talked about a potential surgery
D17
CP: family carer went to pick-up prescription renewal
Cardiologist : follow up for potential surgery
D19
General practitioner: follow-up appointment. GP did not receive documents from hospital
D27-28
CP: The patient had to call the GP to have a new prescription in order to get the medication
Diabetologist : follow up and medication changes
Stop insulin degludec and liraglutide; addition of semaglutide
D39
CP: patient pick-up new medication
GP : new renewable prescription and follow-up of symptoms
D48
D51
Cardiologist: improvement of flutter, no need for surgery

P11

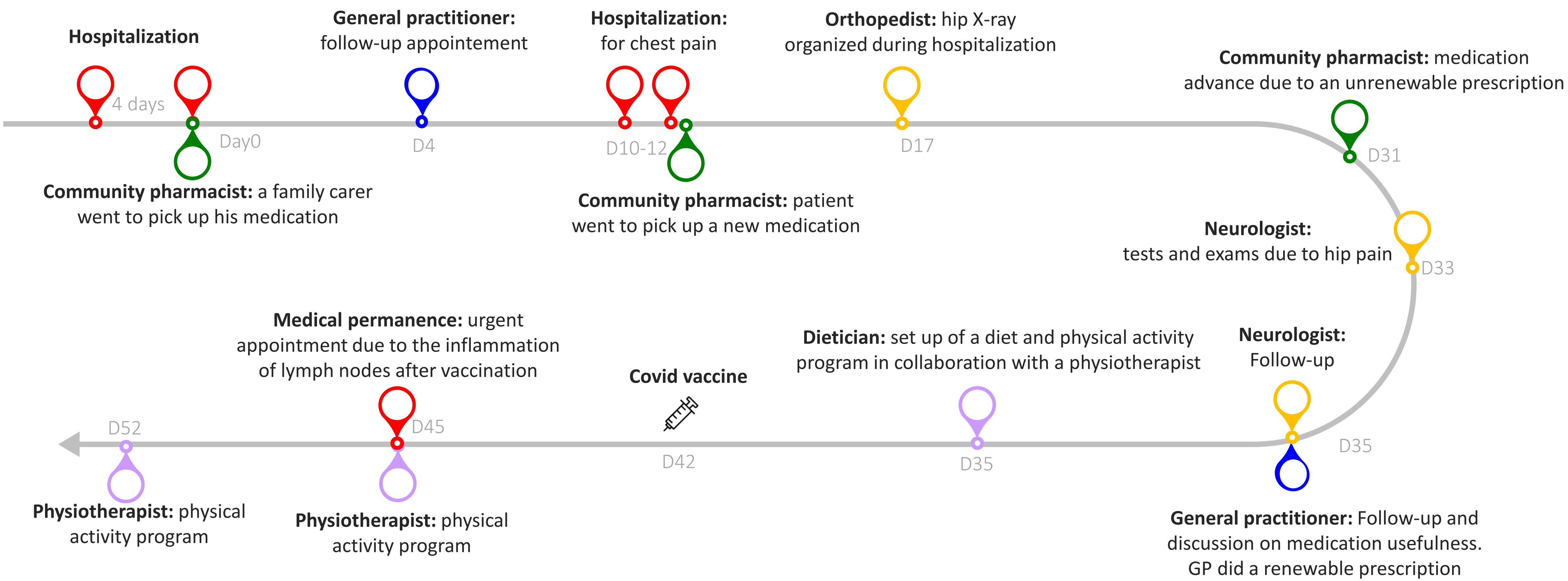

Hospitalization
4 days
Day0
Community pharmacist: a family carer went to pick up his medication
General practitioner: follow-up appointement
D4
Hospitalization: for chest pain
D10-12
Community pharmacist: patient went to pick up a new medication
Orthopedist: hip X-ray organized during hospitalization
D17
Community pharmacist: medication advance due to an unrenewable prescription
D31
Neurologist: tests and exams due to hip pain
D33
Neurologist: Follow-up
D35
General practitioner: Follow-up and discussion on medication usefulness. GP did a renewable prescription
Dietician: set up of a diet and physical activity program in collaboration with a physiotherapist
D35
Covid vaccine
D42
Medical permanence: urgent appointment due to the inflammation of lymph nodes after vaccination
D45
Physiotherapist: physical activity program
D52
Physiotherapist: physical activity program

P12 NEW

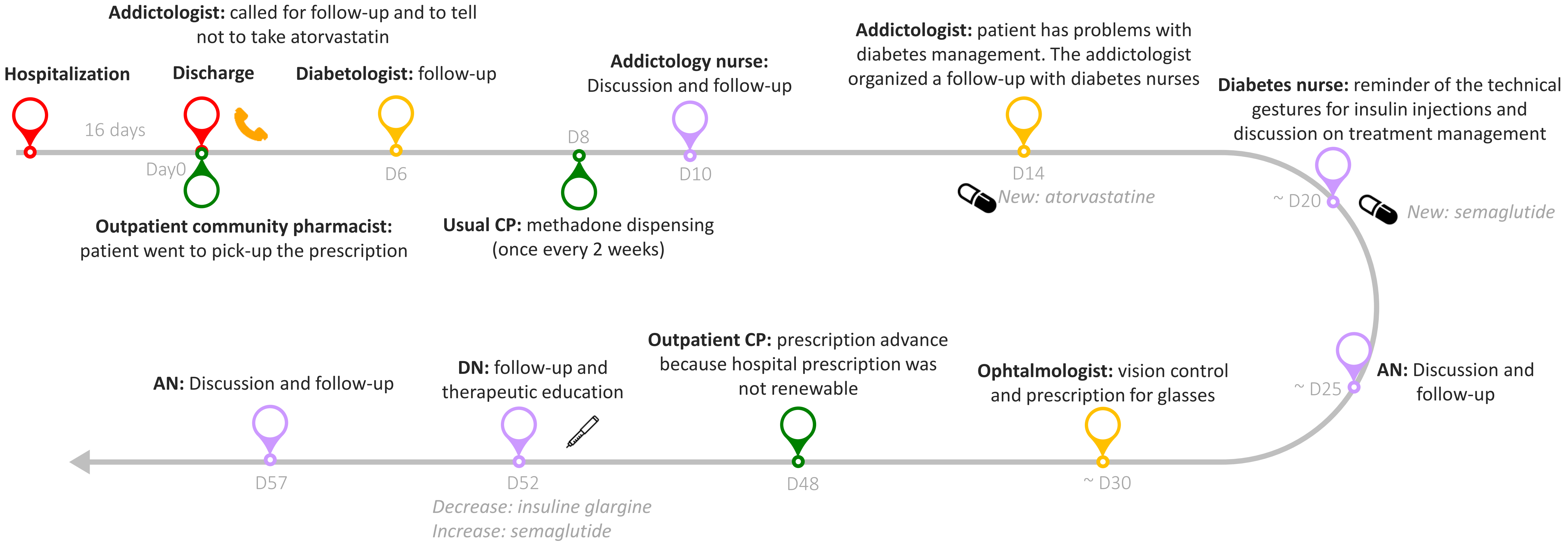
Hospitalization
16 days
Discharge
Day0
Addictologist: called for follow-up and to tell not to take atorvastatin
Outpatient community pharmacist: patient went to pick-up the prescription
Diabetologist: follow-up
D6
D8
Usual CP: methadone dispensing (once every 2 weeks)
Addictology nurse: Discussion and follow-up
D10
Addictologist: patient has problems with diabetes management. The addictologist organized a follow-up with diabetes nurses
D14
New: atorvastatine
Diabetes nurse: reminder of the technical gestures for insulin injections and discussion on treatment management
~ D20
New: semaglutide
~ D25
AN: Discussion and follow-up
Ophtalmologist: vision control and prescription for glasses
~ D30
Outpatient CP: prescription advance because hospital prescription was not renewable
D48
DN: follow-up and therapeutic education
D52
Decrease: insuline glargine
Increase: semaglutide
AN: Discussion and follow-up
D57

P13

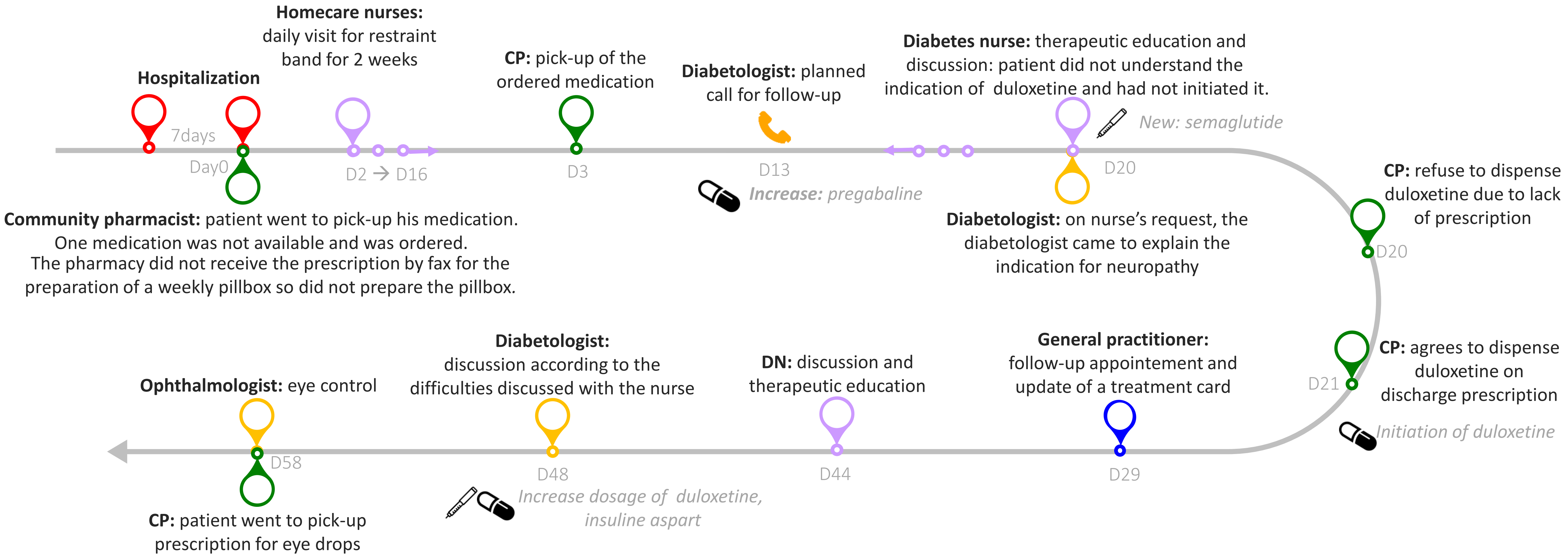
Hospitalization
7days
Day0
Community pharmacist: patient went to pick-up his medication. One medication was not available and was ordered. The pharmacy did not receive the prescription by fax for the preparation of a weekly pillbox so did not prepare the pillbox.
Homecare nurses: daily visit for restraint band for 2 weeks
D2 → D16
CP: pick-up of the ordered medication
D3
Diabetologist: planned call for follow-up
D13
Increase: pregabaline
Diabetes nurse: therapeutic education and discussion: patient did not understand the indication of duloxetine and had not initiated it.
New: semaglutide
D20
Diabetologist: on nurse's request, the diabetologist came to explain the indication for neuropathy
CP: refuse to dispense duloxetine due to lack of prescription
D20
CP: agrees to dispense duloxetine on discharge prescription
D21
Initiation of duloxetine
General practitioner: follow-up appointement and update of a treatment card
D29
DN: discussion and therapeutic education
D44
Diabetologist: discussion according to the difficulties discussed with the nurse
D48
Increase dosage of duloxetine, insuline aspart
Ophthalmologist: eye control
D58
CP: patient went to pick-up prescription for eye drops

P14

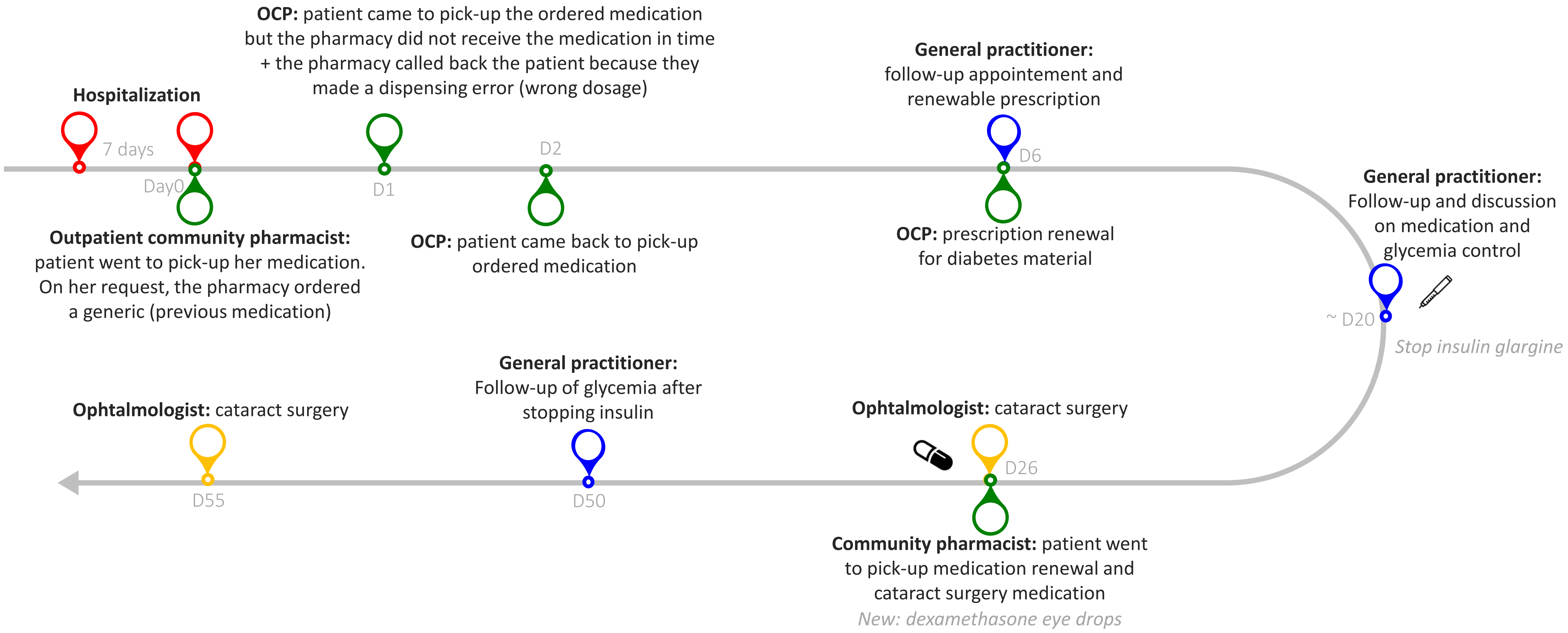
Hospitalization
7 days
Day0
Outpatient community pharmacist: patient went to pick-up her medication. On her request, the pharmacy ordered a generic (previous medication)
OCP: patient came to pick-up the ordered medication but the pharmacy did not receive the medication in time + the pharmacy called back the patient because they made a dispensing error (wrong dosage)
D1
D2
OCP: patient came back to pick-up ordered medication
General practitioner: follow-up appointement and renewable prescription
D6
OCP: prescription renewal for diabetes material
General practitioner: Follow-up and discussion on medication and glycemia control
~ D20
Stop insulin glargine
Ophtalmologist: cataract surgery
D26
Community pharmacist: patient went to pick-up medication renewal and cataract surgery medication
New: dexamethasone eye drops
General practitioner: Follow-up of glycemia after stopping insulin
D50
Ophtalmologist: cataract surgery
D55

P15

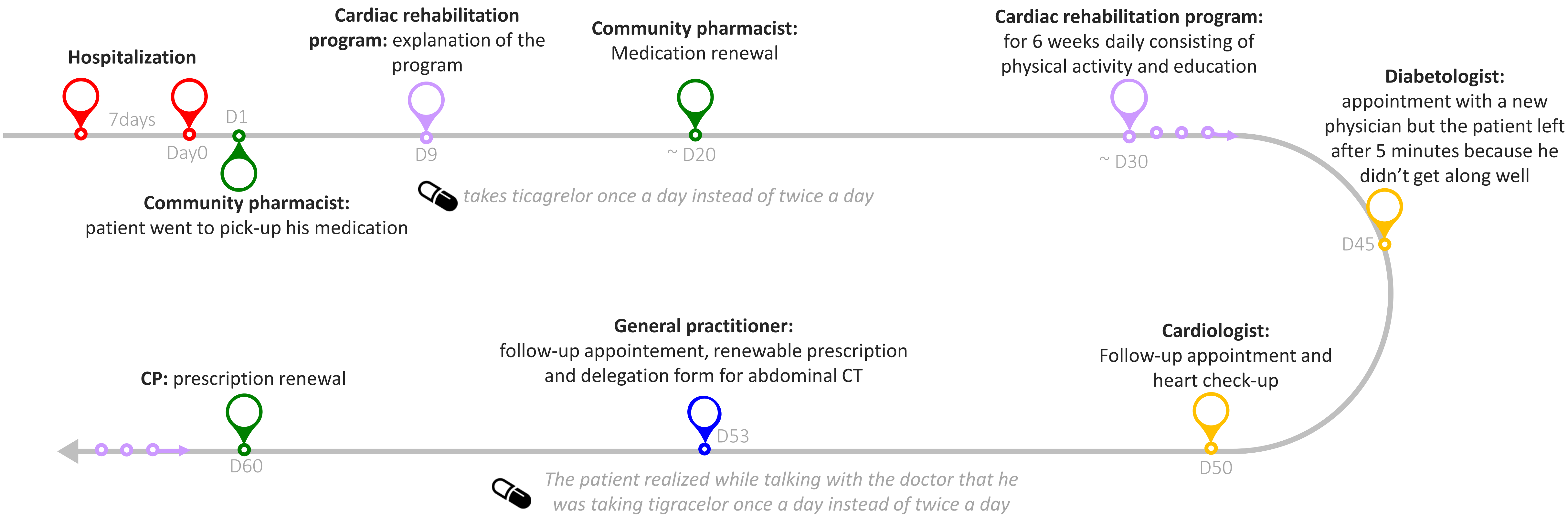
Hospitalization
7days
Day0
D1
Community pharmacist:
patient went to pick-up his medication
Cardiac rehabilitation program: explanation of the program
D9
takes ticagrelor once a day instead of twice a day
Community pharmacist:
Medication renewal
~ D20
Cardiac rehabilitation program:
for 6 weeks daily consisting of physical activity and education
~ D30
Diabetologist:
appointment with a new physician but the patient left after 5 minutes because he didn't get along well
D45
Cardiologist:
Follow-up appointment and heart check-up
D50
General practitioner:
follow-up appointement, renewable prescription and delegation form for abdominal CT
D53
The patient realized while talking with the doctor that he was taking tigracelor once a day instead of twice a day
CP: prescription renewal
D60

P16

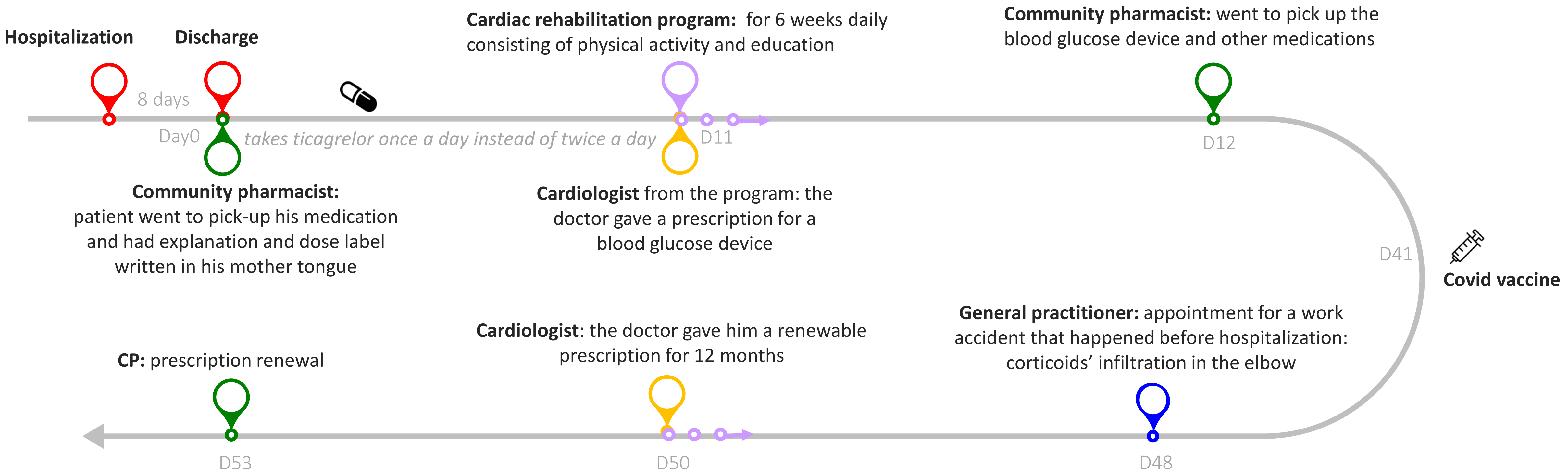
Hospitalization
8 days
Discharge
Day0
Community pharmacist: patient went to pick-up his medication and had explanation and dose label written in his mother tongue
takes ticagrelor once a day instead of twice a day
Cardiac rehabilitation program: for 6 weeks daily consisting of physical activity and education
D11
Cardiologist from the program: the doctor gave a prescription for a blood glucose device
Community pharmacist: went to pick up the blood glucose device and other medications
D12
D41
Covid vaccine
General practitioner: appointment for a work accident that happened before hospitalization: corticoids' infiltration in the elbow
D48
Cardiologist: the doctor gave him a renewable prescription for 12 months
D50
CP: prescription renewal
D53

P17

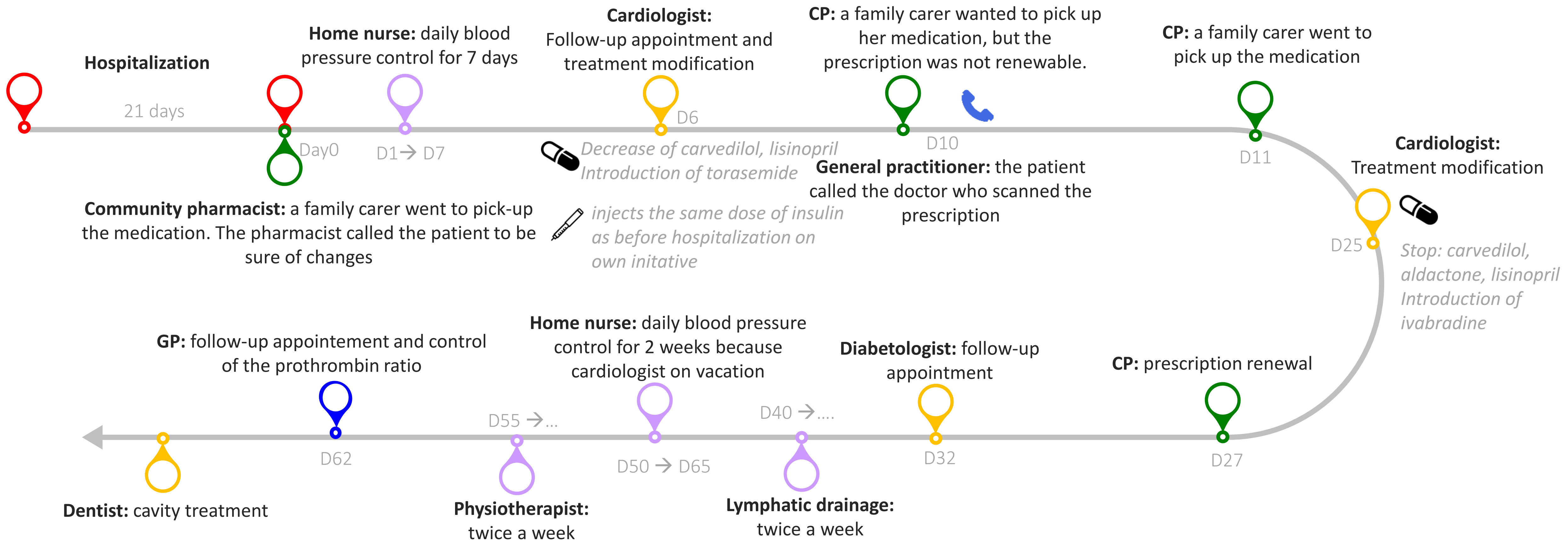
Hospitalization
21 days
Day0
Community pharmacist: a family carer went to pick-up the medication. The pharmacist called the patient to be sure of changes
Home nurse: daily blood pressure control for 7 days
D1→ D7
Cardiologist: Follow-up appointment and treatment modification
D6
Decrease of carvedilol, lisinopril
Introduction of torasemide
injects the same dose of insulin as before hospitalization on own initative
CP: a family carer wanted to pick up her medication, but the prescription was not renewable.
D10
General practitioner: the patient called the doctor who scanned the prescription
CP: a family carer went to pick up the medication
D11
Cardiologist: Treatment modification
D25
Stop: carvedilol, aldactone, lisinopril
Introduction of ivabradine
CP: prescription renewal
D27
Diabetologist: follow-up appointment
D32
D40 →....
Lymphatic drainage: twice a week
Home nurse: daily blood pressure control for 2 weeks because cardiologist on vacation
D50 → D65
D55 →...
Physiotherapist: twice a week
GP: follow-up appointement and control of the prothrombin ratio
D62
Dentist: cavity treatment

P20

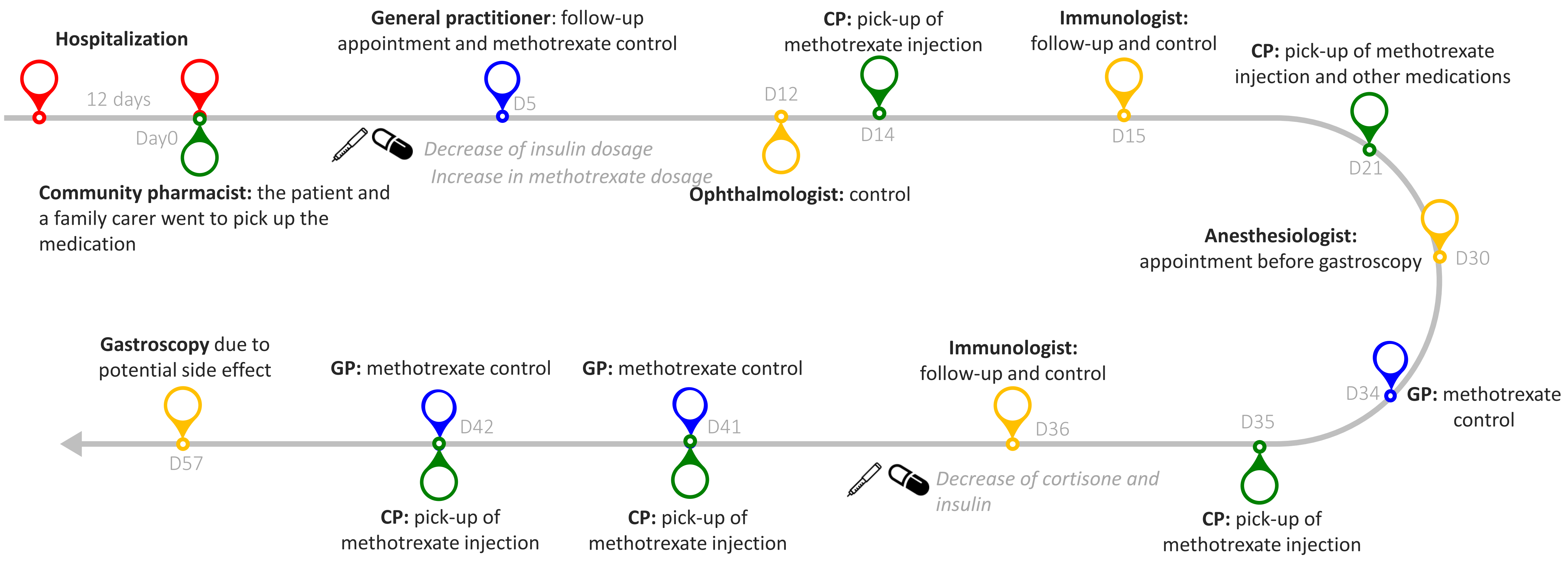
Hospitalization
12 days
Day0
Community pharmacist: the patient and a family carer went to pick up the medication
General practitioner: follow-up appointment and methotrexate control
D5
Decrease of insulin dosage
Increase in methotrexate dosage
D12
Ophthalmologist: control
CP: pick-up of methotrexate injection
D14
Immunologist: follow-up and control
D15
CP: pick-up of methotrexate injection and other medications
D21
Anesthesiologist: appointment before gastroscopy
D30
D34
GP: methotrexate control
D35
CP: pick-up of methotrexate injection
Decrease of cortisone and insulin
Immunologist: follow-up and control
D36
GP: methotrexate control
D41
CP: pick-up of methotrexate injection
GP: methotrexate control
D42
CP: pick-up of methotrexate injection
Gastroscopy due to potential side effect
D57

P21

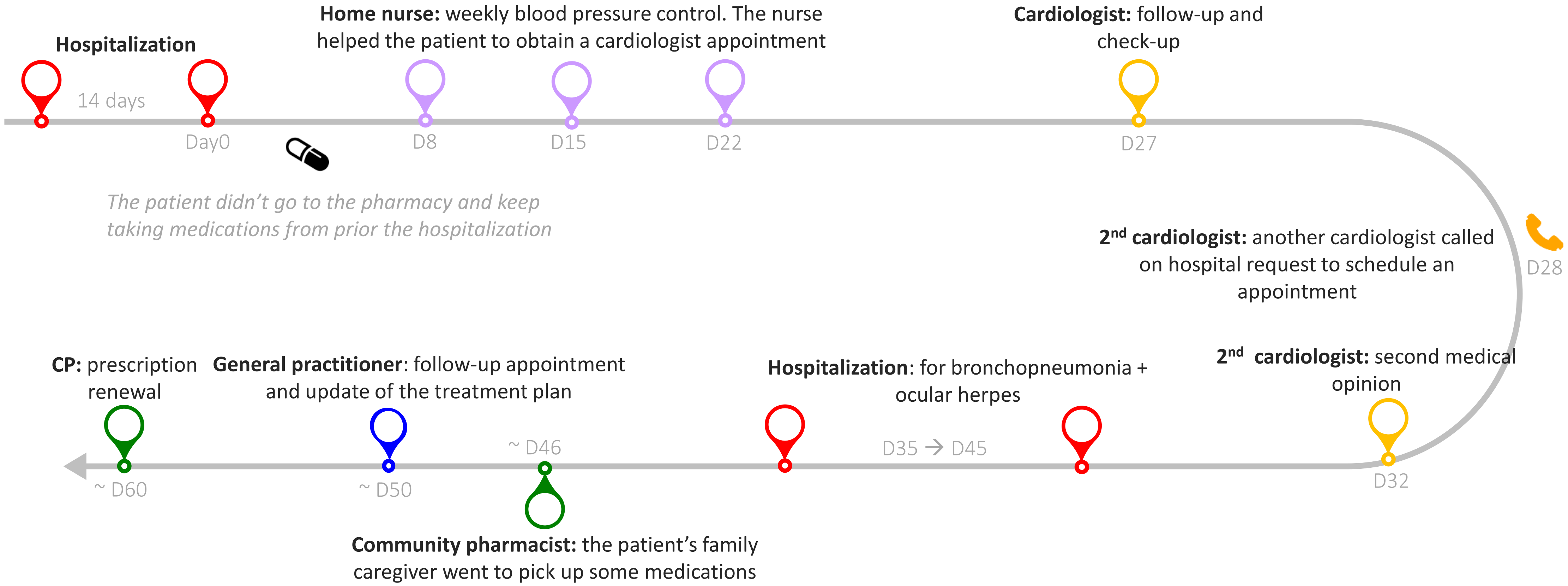
Hospitalization
14 days
Day0
The patient didn't go to the pharmacy and keep taking medications from prior the hospitalization
Home nurse: weekly blood pressure control. The nurse helped the patient to obtain a cardiologist appointment
D8
D15
D22
Cardiologist: follow-up and check-up
D27
2nd cardiologist: another cardiologist called on hospital request to schedule an appointment
D28
2nd cardiologist: second medical opinion
D32
Hospitalization: for bronchopneumonia + ocular herpes
D35 → D45
~ D46
Community pharmacist: the patient's family caregiver went to pick up some medications
General practitioner: follow-up appointment and update of the treatment plan
~ D50
CP: prescription renewal
~ D60

P22

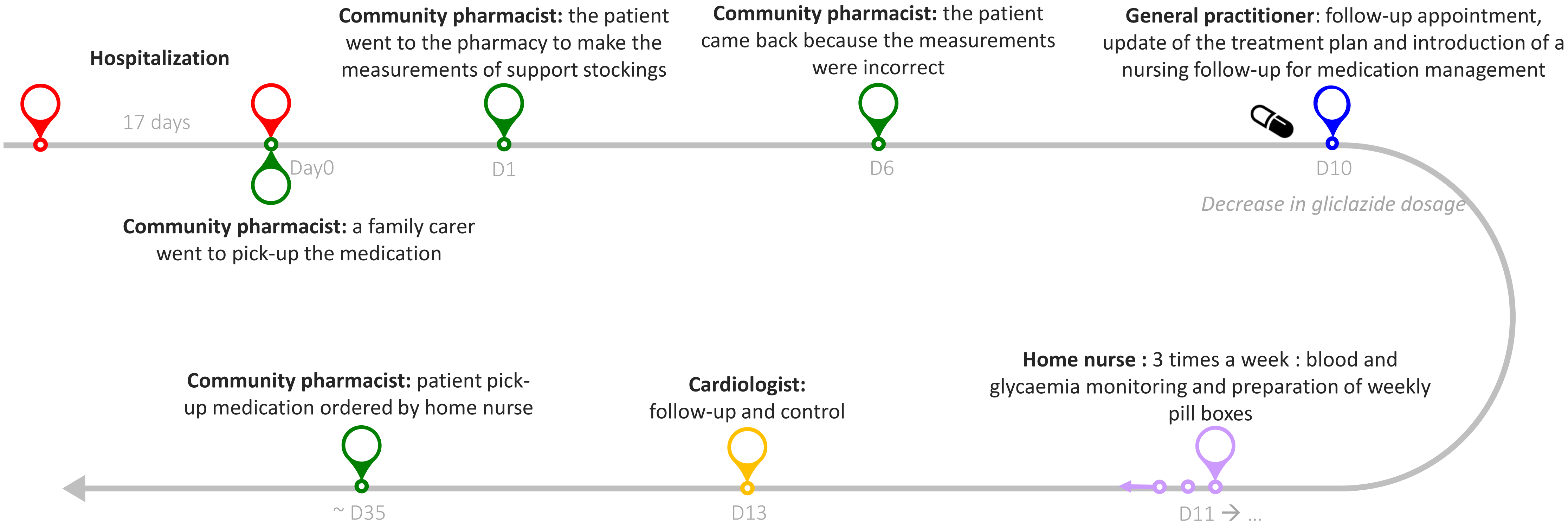
Hospitalization
17 days
Day0
Community pharmacist: a family carer went to pick-up the medication
Community pharmacist: the patient went to the pharmacy to make the measurements of support stockings
D1
Community pharmacist: the patient came back because the measurements were incorrect
D6
General practitioner: follow-up appointment, update of the treatment plan and introduction of a nursing follow-up for medication management
D10
Decrease in gliclazide dosage
Home nurse : 3 times a week : blood and glycaemia monitoring and preparation of weekly pill boxes
D11 → ...
Cardiologist: follow-up and control
D13
Community pharmacist: patient pick-up medication ordered by home nurse
~ D35

P23

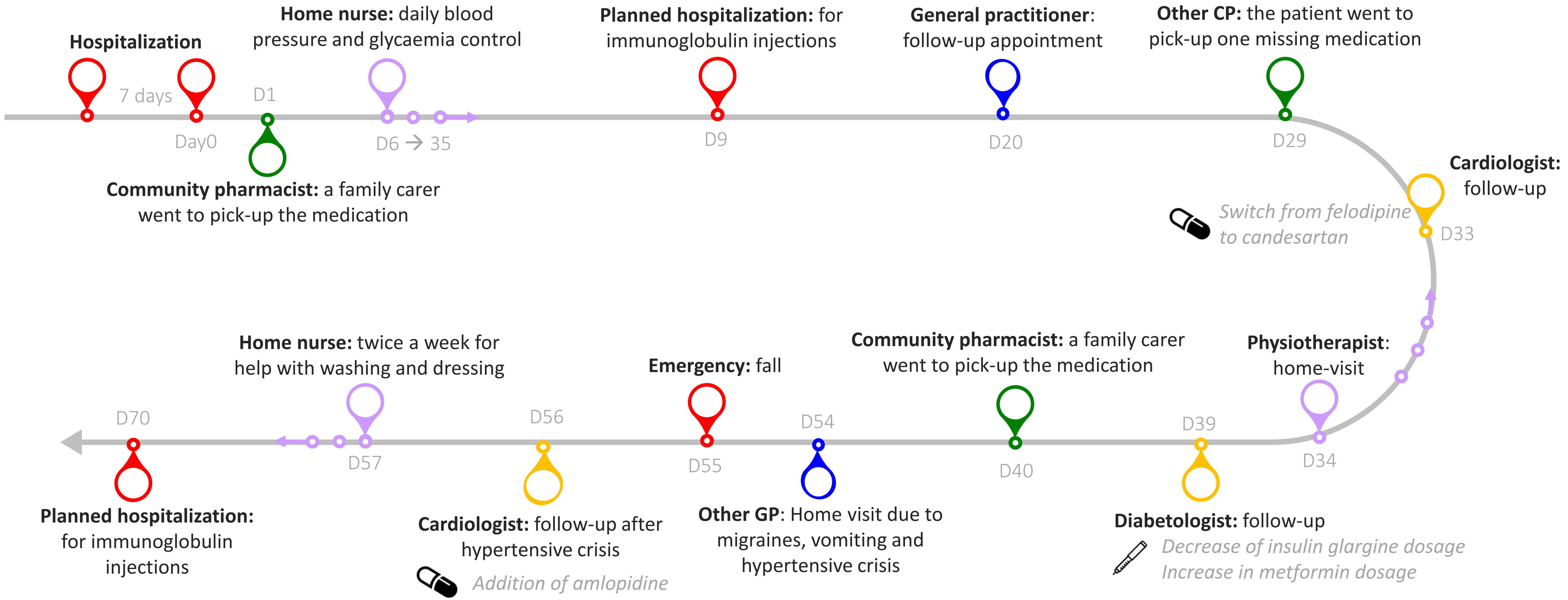

Hospitalization
7 days
Day0
D1
Community pharmacist: a family carer went to pick-up the medication
Home nurse: daily blood pressure and glycaemia control
D6 → 35
Planned hospitalization: for immunoglobulin injections
D9
General practitioner: follow-up appointment
D20
Other CP: the patient went to pick-up one missing medication
D29
Cardiologist: follow-up
D33
Switch from felodipine to candesartan
Physiotherapist: home-visit
D34
Diabetologist: follow-up
D39
Decrease of insulin glargine dosage
Increase in metformin dosage
Community pharmacist: a family carer went to pick-up the medication
D40
Other GP: Home visit due to migraines, vomiting and hypertensive crisis
D54
Emergency: fall
D55
Cardiologist: follow-up after hypertensive crisis
D56
Addition of amlopidine
Home nurse: twice a week for help with washing and dressing
D57
Planned hospitalization: for immunoglobulin injections
D70
